# Peroxidase Activity of Myoglobin Variants Reconstituted with Artificial Cofactors

**DOI:** 10.1002/cbic.202200197

**Published:** 2022-07-28

**Authors:** Chao Guo, Robert J. Chadwick, Adam Foulis, Giada Bedendi, Andriy Lubskyy, Kyle J. Rodriguez, Michela M. Pellizzoni, Ross D. Milton, Rebecca Beveridge, Nico Bruns

**Affiliations:** ^1^ Department of Pure and Applied Chemistry University of Strathclyde 295 Cathedral Street G1 1XL Glasgow UK; ^2^ Department of Inorganic and Analytical Chemistry University of Geneva 1211 Geneva 4 Switzerland; ^3^ Adolphe Merkle Institute University of Fribourg Chemin des Verdiers 4 1700 Fribourg Switzerland; ^4^ Department of Chemistry Technical University of Darmstadt Alarich-Weiss-Str. 4 64287 Darmstadt Germany

**Keywords:** metalloproteins, mutagenesis, myoglobin, peroxidases, reconstitution

## Abstract

Myoglobin (Mb) can react with hydrogen peroxide (H_2_O_2_) to form a highly active intermediate compound and catalyse oxidation reactions. To enhance this activity, known as pseudo‐peroxidase activity, previous studies have focused on the modification of key amino acid residues of Mb or the heme cofactor. In this work, the Mb scaffold (apo‐Mb) was systematically reconstituted with a set of cofactors based on six metal ions and two ligands. These Mb variants were fully characterised by UV‐Vis spectroscopy, circular dichroism (CD) spectroscopy, inductively coupled plasma mass spectrometry (ICP‐MS) and native mass spectrometry (nMS). The steady‐state kinetics of guaiacol oxidation and 2,4,6‐trichlorophenol (TCP) dehalogenation catalysed by Mb variants were determined. Mb variants with iron chlorin e6 (Fe−Ce6) and manganese chlorin e6 (Mn−Ce6) cofactors were found to have improved catalytic efficiency for both guaiacol and TCP substrates in comparison with wild‐type Mb, i. e. Fe‐protoporphyrin IX‐Mb. Furthermore, the selected cofactors were incorporated into the scaffold of a Mb mutant, swMb H64D. Enhanced peroxidase activity for both substrates were found via the reconstitution of Fe−Ce6 into the mutant scaffold.

## Graphical abstract

**To enhance the peroxidase** activity of myoglobin, the effect of artificial cofactors (metal centre and the porphyrin ligand) was investigated. Twelve artificial cofactors were reconstituted with the myoglobin scaffold and FeCe6‐Mb was identified to have 3 times improved catalytic efficiency.

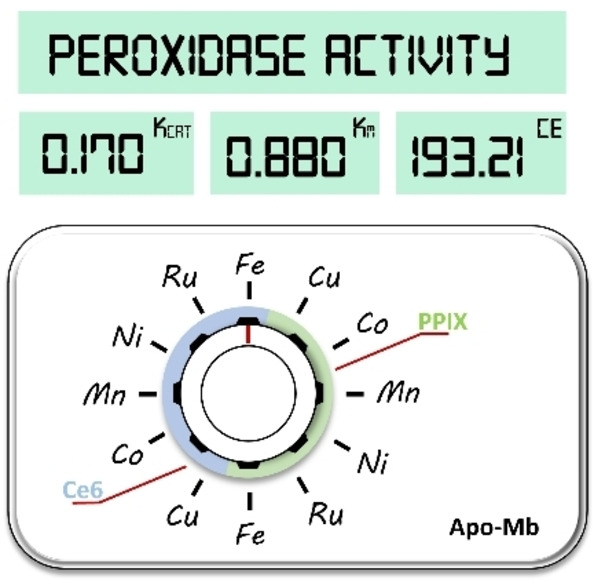

## Introduction

Peroxidases (POD, EC 1.11.1.7) are widely distributed in nature and can be produced by plenty of organsims, including plants, animals and bacteria. These enzymes are activated by hydrogen peroxide (H_2_O_2_) and then catalyse the oxidation of organic and inorganic substrates. PODs are often extracted from their natural source and applied in industrial applications. Examples include the bioremediation of wastewater, biobleaching in paper production and biodegradation of dye and lignin.^[1]^ The use of POD from different sources offers a solution for the degradation of a broad range of environmental pollutants, for example herbicides and pesticides.^[2]^ In the biomedical and biotechnological fields, purified and well‐designed POD are often employed in bioanalytical assays, such as enzyme‐linked immunosorbent assays (ELISA).^[3]^ Horseradish peroxidase (HRP) is the most well‐studied POD. Due to its relatively low molecular weight (44 kDa), high catalytic efficiency and the presence oflysine residues accessible for chemical conjugation, HRP has been used as the reporting enzyme conjugated to antibodies and other biomolecules for qualitative detections.^[4]^ By immobilising HRP on the surface of electrodes, highly sensitive biosensors can be constructed for detection of compounds such as H_2_O_2_, with high specificity.^[5]^

HRP has been shown to be an efficient oxidising enzyme in several types of biotransformation reactions, including hydroxylations, sulfoxidations, coupling reactions and polymerisations.^[6]^ The application of HRP in enzymatic atom transfer radical polymerisations (bioATRP) is a good example for enzymes as tools in green chemistry.^[7]^ Compared to conventional polymerisation, which are often conducted in harsh conditions with toxic reagents, HRP performed these polymerisations under mild conditions. HRP has also been employed to synthesize polyanilines^[8]^ and to initiate reversible addition fragmentation chain transfer (RAFT) polymerisations,^[9]^ which allows for a greener synthesis of well‐defined polymers.

**Scheme 1:**
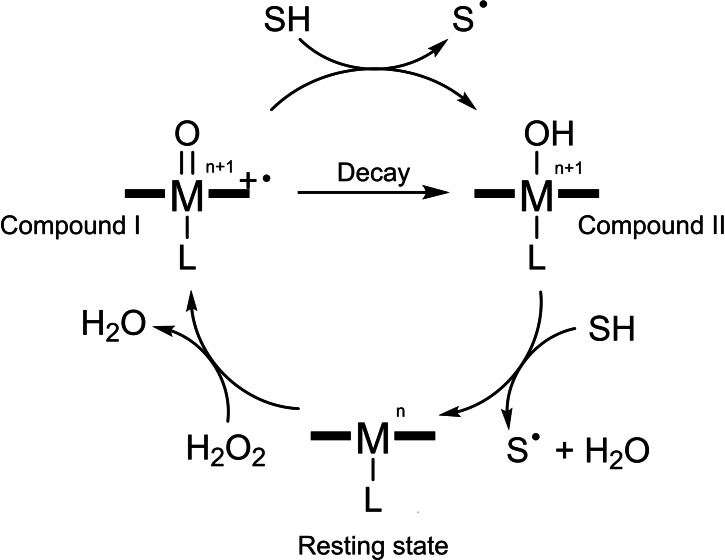
Reaction cycle of Mb as peroxidase. In wild‐type Mb, M^n^=Fe^3+^, SH=substrate, compound I with radical on the heme, Tyr or Trp.

The advantages of metalloenzyme catalysis, such as with HRP, have led to an increased interest in their diverse catalytic abilities and tuneable aspects of the active metal centre.^[10]^ To understand the relationship between structure and functionality, various proteins have been used as models for further investigation and engineering. Among them, myoglobin (Mb) attracted the most attention, given its low molecular weight (17 KDa), known structure and accessible active site.^[11]^ Native Mb can react with H_2_O_2_, forming a highly active compound II (ferryl species Fe^IV^−OH), which then oxidises one equivalent substrate and brings the iron back to the resting state (Scheme 1).^[12]^ Compared to HRP,^[13]^ compound I (ferryl species Fe^IV^=O with a heme or protein radical cation) is very short‐lived in the Mb reaction cycle and hard to observe. As there is only one oxidising equivalent, the peroxidase activity of myoglobin is lower than of other peroxidases. To overcome this disadvantage, site‐directed mutagenesis has been applied to modify several key amino acid sites. One successful mutant, Mb H64D, promoted the formation and stabilisation of compound I.^[14]^ As a result, this mutant showed significantly increased activities in guaiacol oxidation and thioanisole sulfoxidation.^[14]^ Another approach to tune the peroxidase activity of Mb was recently reported by Pott and co‐workers.^[15]^ They targeted the conserved proximal site (H93) of Mb and replaced it with noncanonical N_δ_‐methyl histidine. The generated mutant had an increased heme redox potential and increased catalytic efficiency.

To modify the electronic environment of the metal centre, an alternative method is to replace the cofactor of Mb with an artificial one. The iron of heme in myoglobin has been replaced with a variety of metal ions, including manganese^[16]^ and ruthenium.^[17]^ The Hayashi group has reported several artificial Mbs by reconstitution of chemically modified heme and replacement of porphyrin with other macrocycles.^[18]^ The Fasan^[19]^ and Hartwig^[20]^ groups have also developed Mb catalysts with artificial noble metal cofactors. They have focused on the capability of Mbs as catalysts for carbene transfer reactions, functionalization of C−H bonds and other asymmetric syntheses.

To date, there is no systematic comparison between a set of cofactors reconstituted within the Mb scaffold. Moreover, except for iron chlorin e6 myoglobin (FeCe6‐Mb),^[19e]^ other Ce6‐containing Mb variants have not been reported. Our group has recently applied the cofactor copper Ce6 as a catalyst in atom transfer radical polymerisations (ATRP).^[21]^ Compared to the native protoporphyrin IX (PPIX), Ce6 contains an unsaturated pyrrole ring and an extra carboxylic group (see Scheme S1 for the structures). Ce6 might create a more electron‐deficient environment to the metal centre and form a stronger oxidising compound II for oxidation reactions when reconstituted in Mb. As the central metal ion also affects the redox potential and planar structure of cofactor, the native cofactor of Mb was replaced with two sets of artificial cofactors based on PPIX and Ce6. Their effect on the reconstitution efficiency and catalytic activities of Mb variants were analysed.

## Results and Discussion

Six metal ions, namely iron(III), copper(III), cobalt(III), nickel(II), manganese(III) and ruthenium(II), were inserted into two types of ligands (PPIX and Ce6) and a cofactor library containing twelve artificial cofactors was constructed. The successful insertion of the metal ions into the porphyrin ligands was confirmed by UV‐Vis spectroscopy and mass spectrometry. In absence of metal ions, the two porphyrins showed different absorbance in the Soret band and Q band (Figure S1). Compared to the PPIX, the Q band of Ce6 was shifted to a shorter wavelength with higher intensity because one β‐position of Ce6 is directly attached to a carboxylic acid group. After the insertion of metal ions, all Q bands of the macrocycles were simpler with two obvious peaks, which was a result of the symmetric geometry of the complexes (Figure S2, S3 and Table S1). The Mn‐PPIX and Mn−Ce6 were exception here, as their Soret bands were split into two relatively weak bands, rather than the normal intense single band. The anomalous spectrums of Mn‐PPIX and Mn−Ce6 could be related to strong π mixing between the metal anion and the porphyrins.^[22]^

**Figure 1:**
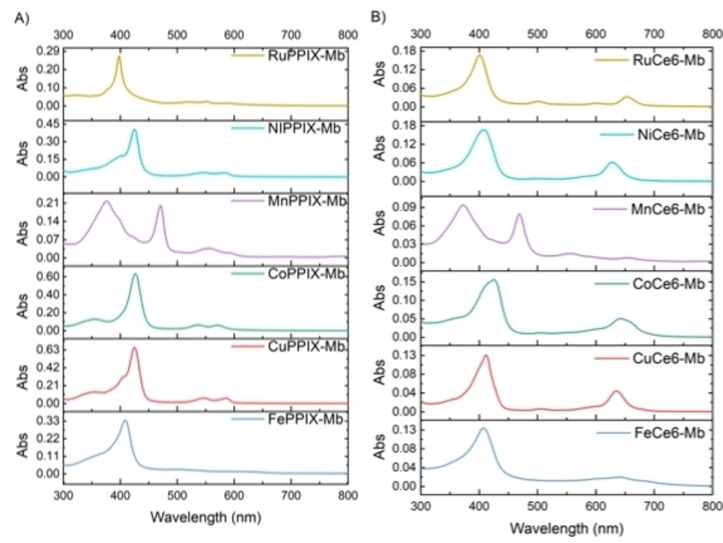
UV‐vis spectra of Mb variants with A) reconstituted PPIX cofactors and B) with Ce6 cofactors. Protein samples (4 nM) were measured in PBS buffer (pH 7.4) at 22 °C.

Apo‐Mb was prepared based on Teale's method.^[23]^ The protein scaffold was obtained from commercially available equine skeletal muscle Mb after the native heme cofactor was extracted by 2‐butanone in acidic conditions. The artificial cofactors were reconstituted in the protein scaffold separately. The native heme (Fe‐PPIX) was re‐inserted into the apo‐Mb as the benchmark wild‐type, which we name here FePPIX‐Mb. The generated Mb variants were characterized by UV‐Vis spectroscopy, sodium dodecyl sulfate polyacrylamide gel electrophoresis (SDS‐PAGE), inductively coupled plasma mass spectrometry (ICP‐MS) and native mass spectrometry (nMS). The UV‐Vis spectra of Mb variants were distinguishable from each other (Figure 1). All the Soret bands of Mb variants were shifted to a longer wavelength in comparison with their free cofactors owing to the formation of a coordinate‐covalent bond between the histidine (H93) and metal centre (Tables S1 and S2). The absorbance of Soret bands were correlated with the concentration of metal ions determined by ICP‐MS to calculate the molar extinction coefficients (ϵ) of Mb variants (Table S2). Based on these values, catalysis experiments were carried out with a defined enzyme concentration. The measured extinction coefficients are in agreement with Soret extinction coefficients that have been reported previously (Table S2), which can be taken as validation of the employed methods. One exception is FeCe6‐Mb, where the extinction coefficient that we measured is approx. 60 % of the one reported in literature. While this could be due to partial cofactor reconstitution, the mass spectrometry data discussed below and summarized in Table S3 indicates a complete cofactor incorporation for this variant.

In SDS‐PAGE (Figure S4), all Mb variants showed a single band around 17 kDa, which indicates that the protein scaffold of the Mb variants were maintained well without degradation. Circular dichroism (CD) spectroscopy was employed to help confirm the secondary structure of the Mb variants (Figure S5). Compared to FePPIX‐Mb, apo‐Mb lost about 20 % of its α‐helices.^[24]^ After reconstitution with cofactors, the α‐helices were mostly recovered. The variants also share a similar ratio of β‐strands to turn structures with the FePPIX‐Mb. These results indicate that the artificial metalloproteins have similar secondary structure than the native Mb.

**Figure 2:**
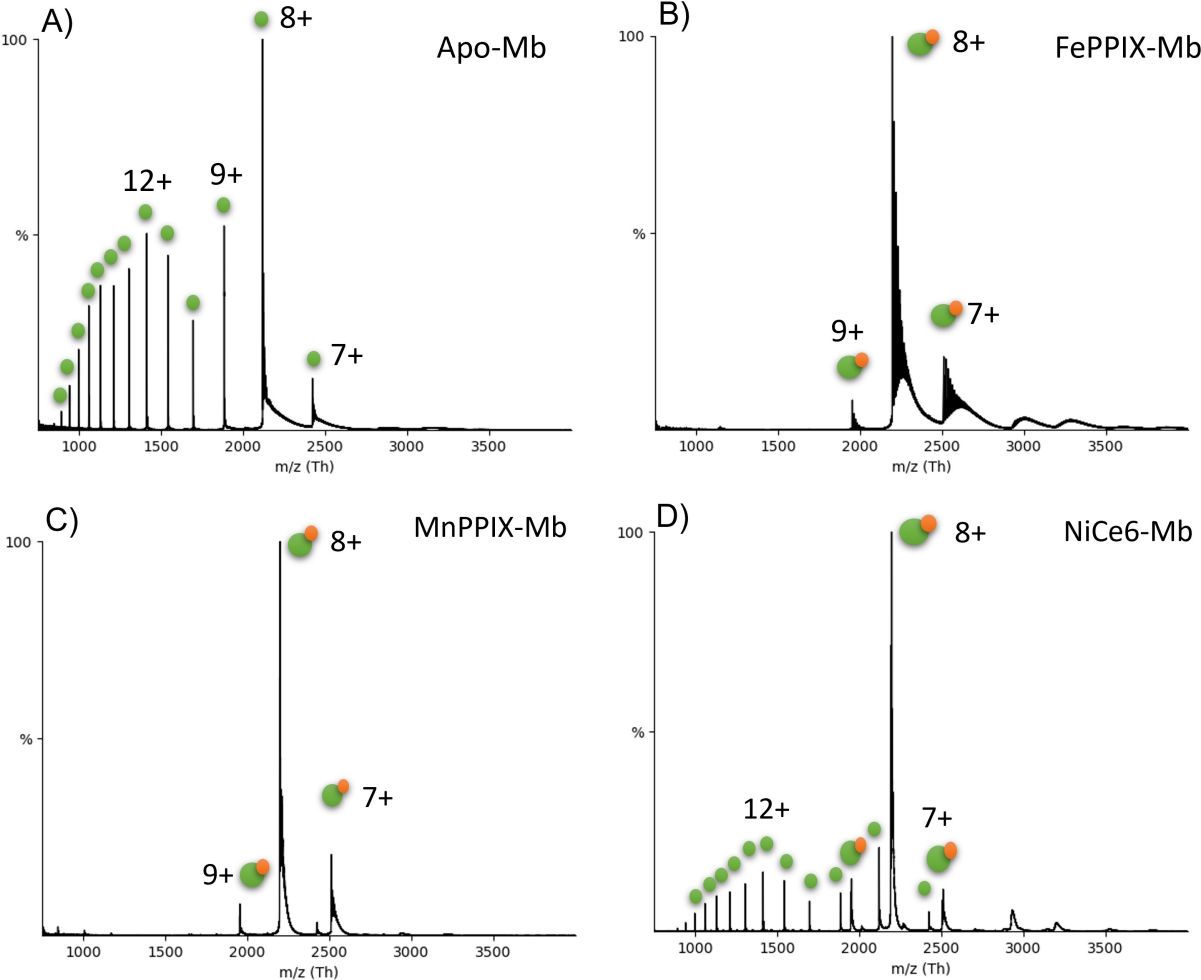
Native mass spectra of apo‐Mb and selected Mb variants. A) shows the nMS spectrum of apoMb with wide range of charge states. B), C) show the spectra of FePPIX‐Mb and MnPPIX‐Mb, the charge states are from 7+ to 9+. D) shows the spectrum of NiCe6‐Mb where the protein is present in both its bound and unbound forms. The bound complex (orange on green symbol) has low charge states, indicating compaction, while the unbound protein (green symbol) has higher charge states, indicating denatured conformations.

Native mass spectrometry confirmed that all the artificial cofactors were inserted into the scaffold of apo‐Mb (Figure 2, Figure S6, and Table S3). Mb variants such as FePPIX‐Mb and MnPPIX‐Mb (Figure 2 B and C) were in their fully reconstituted forms, and had low charge states of 7+, 8+ and 9+, indicating that the cofactors stabilised the scaffold of Mb and that the protein complexes had compact conformations.^[25]^ However, the nMS spectra of four variants (NiPPIX‐Mb, NiCe6‐Mb, MnCe6‐Mb and RuCe6‐Mb) showed signal corresponding to Mb in both its bound and unbound form. This could be due the incomplete reconstitution of these artificial cofactors into apo‐Mb, or that these cofactor‐protein complexes were unstable and gradually unbound.

The redox potential of FeCe6‐Mb was identified as +74±3 mV (vs SHE), which was 31 mV more positive than FePPIX‐Mb (+43±4 mV vs SHE) (Figures S7 and S8). This result supports the hypothesis mentioned previously that the electron‐deficient feature of Ce6 would increase the redox potential of the Mb variant.

Before testing the peroxidase activity of all the Mb variants, the reaction conditions were optimised with FePPIX‐Mb. Two substrates, *o*‐methoxyphenol (guaiacol) and 2,4,6‐trichlorophenol (TCP), were found to be ideal test cases given their colorimetric oxidation reactions. All the peroxidase reactions were carried out in PBS buffer (0.1 M, pH 7.4).^[26]^ For the guaiacol oxidation reactions, the concentration of Mb was 2 μM and the guaiacol concentration was 5 mM. The concentration of H_2_O_2_ was then varied from 10 to 200 mM. It was found that 100 mM H_2_O_2_ resulted in the highest initial reaction rate (*V*_0_) (Figure S9). For the TCP dehalogenation reaction, the concentration of Mb and of TCP was set to 1 μM and 1 mM, respectively. The concentration of H_2_O_2_ was varied from 2 to 20 mM. The reaction rate *V*_0_ was highest at 10 mM H_2_O_2_ (Figure S9). These results are consistent with previous findings that higher concentration of hydrogen peroxide can oxidise compound II and inhibit the peroxidase activity.^[27]^

The activity of all the Mb variants was screened based on the optimized reaction conditions (Table S4). FePPIX‐Mb, FeCe6‐Mb, CuCe6‐Mb and MnCe6‐Mb showed activity in both guaiacol and TCP oxidation reactions. MnPPIX‐Mb was found to only catalyse the dehalogenation reaction of the TCP substrate, but not the guaiacol reaction. Other variants, including CuPPIX‐Mb, CoPPIX‐Mb, CoCe6Mb, NiPPIX‐Mb, NiCe6‐Mb, RuPPIX‐Mb and RuCe6‐Mb were found to be inactive in both guaiacol oxidation and TCP dehalogenation reactions.

**Figure 3:**
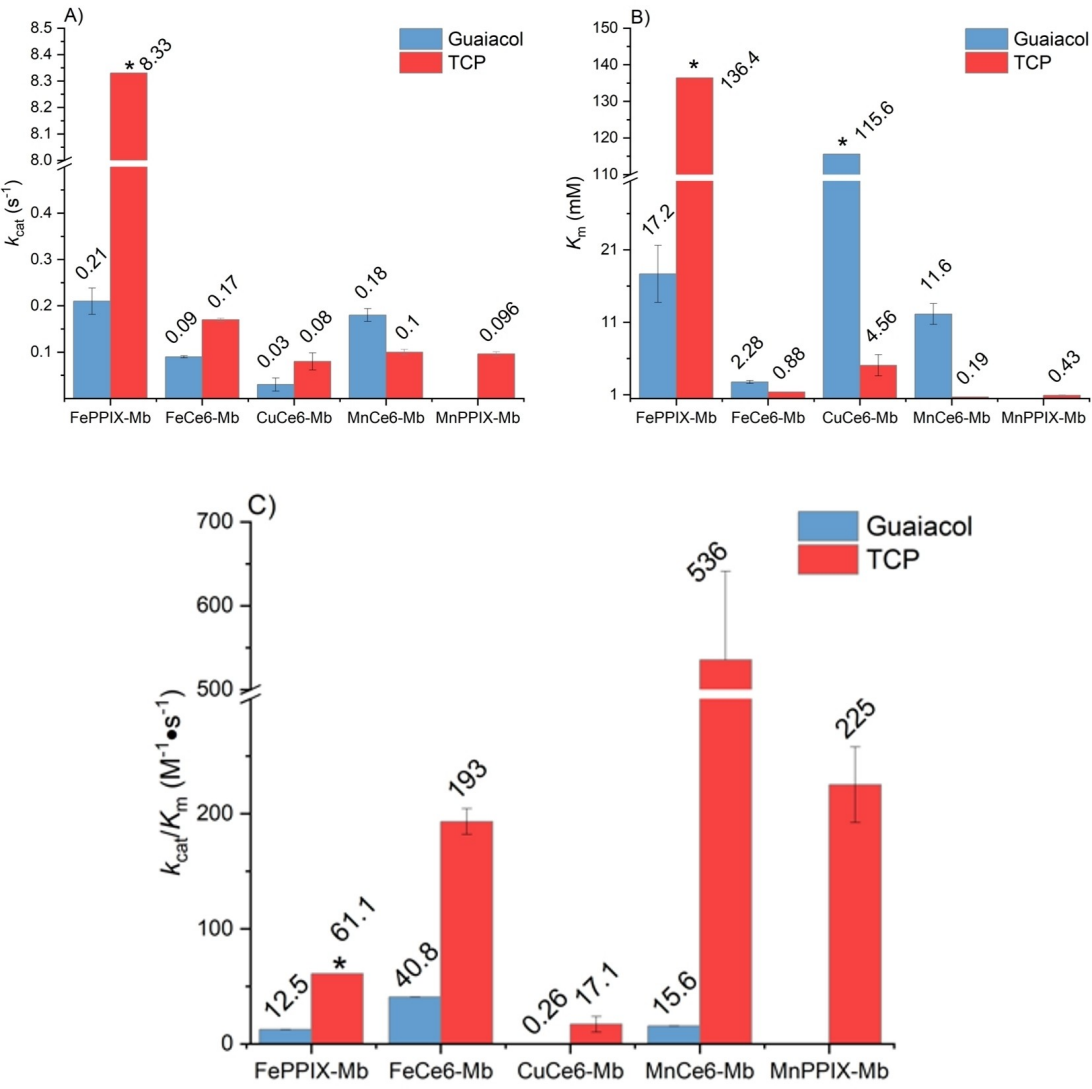
The *k*_cat_, *K*_M_ and catalytic efficiency of Mb variants in peroxidase reactions. A) shows the *k*_cat_, B) shows the *K*_M_ and C) shows the catalytic efficiency (*k*_cat_/*K*_M_) of Mb variants in H_2_O_2_‐dependent oxidation of guaiacol and dehalogenation of TCP. Reaction conditions: guaiacol oxidation, 100 mM H_2_O_2_ and variable [guaiacol]. TCP oxidation, 10 mM H_2_O_2_ and variable [TCP]. All reactions were carried out in PBS buffer (pH 7.4) at 22 °C. Mean values and standard deviations of triplicates are shown. * In these experiments, substrate concentration could not be increased to reach full saturation of the active site before visible protein precipitation occurred. Therefore, the fitting of the kinetic data to obtain *K*_M_ and *k_cat_* resulted in the estimates reported here with large error margins.

Kinetic parameters were determined for the reactive variants with varied concentration of substrates and a constant concentration of H_2_O_2_ (Figures 3, S10 and S11, Table S5 and S6). In the guaiacol oxidation reactions, FeCe6‐Mb and MnCe6‐Mb showed higher affinity to the substrate than FePPIX‐Mb, as the *K*_M_ values dropped to 2.3 mM and 11.6 mM, compared to 17.2 mM of FePPIX‐Mb. In contrast, the *K*_M_ of CuCe6‐Mb was 115.6 mM, which was 6.7‐fold higher than the *K*_M_ of the FePPIX‐Mb. The *k*_cat_ of CuCe6‐Mb, FeCe6‐Mb and MnCe6‐Mb were 0.14‐fold, 0.43‐fold and 0.85‐fold of the *k*_cat_ of FePPIX‐Mb. Thus, under these reaction conditions, FeCe6‐Mb showed three times higher catalytic efficiency (*k*_cat_/*K*_M_) in comparison with FePPIX‐Mb, while the catalytic efficiency of MnCe6‐Mb was 1.25 times higher, and CuCe6‐Mb was 50 times lower.

In the dehalogenation reaction, FePPIX‐Mb showed the lowest affinity to the substrate TCP. During the measurement, even 3 mM TCP did not fully saturate the active site of 0.5 μM FePPIX‐Mb. When a higher concentration of TCP was added to the reaction solution, visible precipitate formed. Thus, it was assumed that the enzyme denatured. Given that it was not possible to increase the substrate concentration further, the fitting of the activity data to obtain a Michaelis‐Menten constant and maximum catalytic activity resulted in large error margins. All the other reactive Mb variants showed higher TCP affinity than FePPIX‐Mb, corresponding to decreased *K*_M_ values. Thus, although the *k*_cat_ values of Mb variants were 50 to 100 times lower than the reconstituted wild‐type Mb, the catalytic efficiency of FeCe6‐Mb, MnCe6‐Mb and MnPPIX‐Mb were all significantly higher than the FePPIX‐Mb with 3.2, 8.8 and 3.7‐times improvements of *k*_cat_/*K*_M_, respectively. The *k*_cat_/*K*_M_ value of CuCe6‐Mb was 3.6 times lower than FePPIX‐Mb, which was caused by the higher *K*_M_ of 4. 6 mM and the approximately 100‐times lower *k*_cat_ value of CuCe6‐Mb.

**Figure 4:**
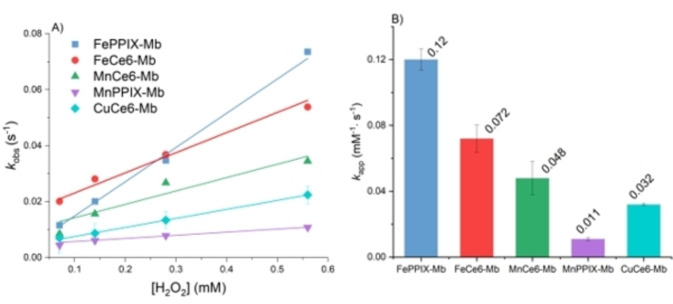
A) Pseudo‐first‐order *k*_obs_ for the formation of compound II when FePPIX‐Mb and Mb variants with reconstituted artificial cofactors reacted with varied [H_2_O_2_]. Mb sample reacted with 40, 20, 10, 5 equiv. H_2_O_2_ in PBS buffer (pH 7.4) at 22 °C. *k*_obs_ was obtained from the decay of Soret peaks with fitted function. B) shows the rate constant (*k*_app_) of compound II formation from plot A). Mean values and standard deviations of triplicates are shown.

The peroxidase cycle of Mb can be mainly divided into two steps, the formation of active Mb species and the transfer of electrons from the substrate to the active species (Scheme 1). The *k*_cat_ parameters mainly describe the reaction rate of latter. To provide more insights into the first step of peroxidase activity, the formation of highly reactive metal‐oxygen intermediates was analysed via stopped‐flow UV‐vis spectroscopy studies. Initially, 20 equiv. H_2_O_2_ were added to react with the Mb variants (Figure S12). The Soret peak of FePPIX‐Mb decreased while a new peak rose around 420 nm (Figures S12 and S13). There were no obvious new peaks generated when FeCe6‐Mb, CuCe6‐Mb, MnCe6‐Mb and MnPPIX‐Mb reacted with H_2_O_2,_ but their Soret peaks decreased during the reactions. In the range from 600 to 700 nm, no transient peaks were detected, which indicates that only compound II was observed. The Soret peaks of other variants did not show detectable changes during the reaction with H_2_O_2_ under the same conditions, indicating that either compound II failed to form or was not observable due to low stability (Figure S12). The decay rate of the Soret peaks of FePPIX‐Mb, FeCe6‐Mb, CuCe6‐Mb, MnCe6‐Mb and MnPPIX‐Mb were measured when incubated with 5, 10, 20 and 40 equivalents of H_2_O_2_. Single exponential functions were fitted to the kinetic traces (Figure S13 and Table S7) and the observed rate constant (*k*_obs_) of compound II formation was calculated from the fits. By plotting the *k*_obs_ versus H_2_O_2_ concentration and linear regression, the apparent rate constants (*k*_app_) for the formation of compound II of the Mb variants were obtained (Figure 4).^[28]^ The *k*_app_ of FeCe6‐Mb was 0.072 mM^−1^ s^−1^, which was 1.5‐fold of MnCe6‐Mb (0.048 mM^−1^ s^−1^), 2.3‐fold of CuCe6‐Mb (0.032 mM^−1^ s^−1^) and 6.5‐fold of MnPPIX‐Mb (0.011 mM^−1^ s^−1^). The *k*_app_ of FePPIX‐Mb was 0.12 mM^−1^ s^−1^, i. e. 1.7 times higher than the highest *k*_app_ of an artificial cofactor variant, FeCe6‐Mb. The smaller *k*_app_ constants of the four artificial cofactor Mb variants explain their decreased reactivity (i. e. *k*_cat_) in peroxidase reactions which can be attributed to unfavourable H_2_O_2_ binding and activation. Ce6 has one extra carboxylic group and one unsaturated pyrrole ring as compared to PPIX, leading to the inserted metal ion having less electron density. This change of electronic properties may trigger the distortion of the Ce6‐based cofactors from planarity and decrease the p*K*_a_ for H_2_O_2_ deprotonation, which then cause inefficient H_2_O_2_ binding and activation.^[29]^

The trend of *k*_app_ values matches the order of *k*_cat_ values for FePPIX‐Mb and the artificial cofactor variants in the guaiacol oxidation reactions, which suggests that the reaction rate of guaiacol oxidation heavily relied on the rate of formation and stability of compound II. In contrast, in the TCP dehalogenations catalysed by MnCe6‐Mb, CuCe6‐Mb and MnPPIX‐Mb, the *k*_cat_ values were close to each other, which indicates that in addition to the effect from compound II formation, the binding of substrate or metal species may affect the reaction rate. It was also noticed that the plot line of FeCe6‐Mb had a higher intercept than others, which could be due to a higher dissociation rate constant of compound II.[^28a^, ^30^]

Another interesting trend was that the *k*_app_ of MnCe6‐Mb was higher than MnPPIX‐Mb, and *k*_app_ of CuCe6‐Mb was higher than CuPPIX‐Mb, which was inactive. This observation shows the advantage of Ce6 as cofactor for Mb, particularly when the metal species was not the native iron. Based on the same ligand and same reaction conditions, the experimental results show that iron was still the most suitable metal ion for peroxidase activities and manganese was slightly better than copper.

The peroxidase activity experiments Mb with native and artificial cofactors discussed above indicate that FeCe6‐Mb and MnCe6‐Mb show better performance in guaiacol oxidation and TCP dehalogenation reactions, given the markedly increased catalytic efficiency compared to FePPIX‐Mb. These two variants were therefore taken forward to further enhance their catalytic performance by protein engineering, more precisely by rational design of the active site.

The distal histidine (H64) is believed to play a major role in the H_2_O_2_ binding and activation. In the reported structure of wild‐type Mb, H64 is in close proximity of the metal centre of the cofactor, limiting its function to donate and accept protons during the peroxidase reaction cycle of Mb. Matsui and co‐workers proved that changing this native histidine into an aspartic acid (D) alleviated the decay rate of compound I into compound II.^[14]^ A longer‐lived compound I and a suitable proton transfer microenvironment increased the peroxidase activity of Mb. They attributed this positive effect to the tuned polarity, which raised the affinity of H_2_O_2_ to the active pocket. Also, the side chain of Asp could form a hydrogen‐bond network with several water molecules in the distal pocket which then function as a water channel for solvent access to the catalytic centre.^[31]^

**Figure 5:**
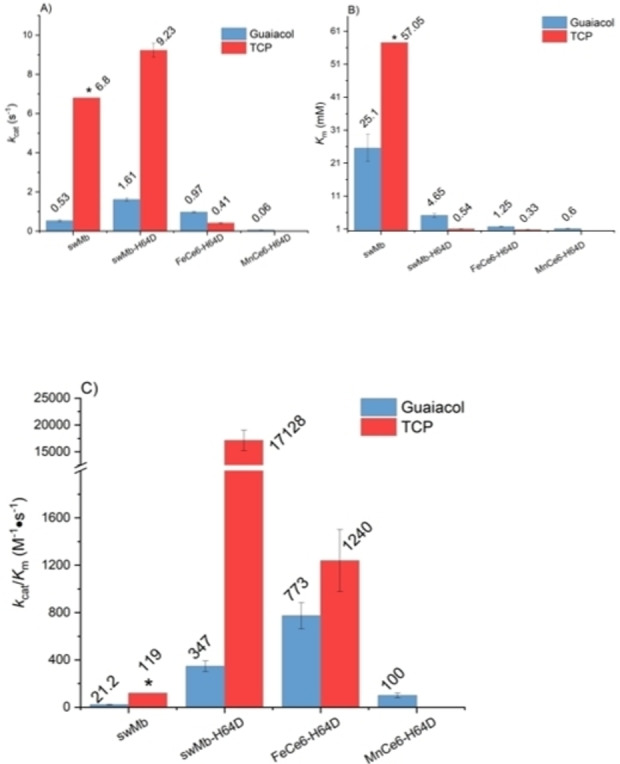
The *k*_cat_, *K*_M_ and catalytic efficiency of swMb‐H64D variants A) shows the *k*_cat_ of Mb variants in H_2_O_2_‐dependent oxidation reactions on guaiacol and TCP. B) shows the *K*_M_ of Mb variants in H_2_O_2_‐dependent oxidation reactions on guaiacol and TCP. C) shows the catalytic efficiency (*k*_cat_/*K*_M_) of Mb variants in H_2_O_2_‐dependent oxidation reactions on guaiacol and TCP. Reaction conditions: guaiacol oxidation, 100 mM H_2_O_2_ and variable [guaiacol]. TCP oxidation, 10 mM H_2_O_2_ and variable [TCP]. All reactions were carried out in PBS buffer (pH 7.4) at 22 °C. Mean values and standard deviations of triplicates are shown. * In these experiments, substrate concentration could not be increased to reach full saturation of the active site before visible protein precipitation occurred. Therefore, the fitting of the kinetic data to obtain *K*_M_ and *k_cat_* resulted in the estimates reported here with large error margins.

To elucidate the effect of active site engineering on the performance of the artificial cofactor myoglobins, it was decided to use a Mb H64D mutant as scaffold. Sperm whale myoglobin (swMb) shares 88 % identity to commercial (equine skeletal muscle) Mb and has similar folding behaviour.^[32]^ More importantly, swMb can be readily expressed via a synthetic gene in *E. coli*, while equine skeletal muscle Mb is less stable and tends to aggregate at higher concentration when recombinantly expressed.^[33]^ Wild type swMb and swMb‐H64D was therefore expressed and purified. Following the same procedure as detailed above, the Fe‐Ce6 and Mn‐Ce6 cofactors were reconstituted within these apo‐proteins. swMb, swMb‐H64D, FeCe6‐swMb‐H64D and MnCe6‐swMb‐H64D were then used to catalyse the reactions of guaiacol and TCP (Figure 5 and Table S6). The kinetic parameters, especially the *k*_cat_ values, of swMb and commercial Mb (from horse skeletal muscle) were close, which can be explained by the high structural similarity of both types of Mb. swMb‐H64D had a significantly increased catalytic efficiency in the oxidation of guaiacol (16.4‐fold increase of *k*_cat_/*K*_M_) and TCP (144‐fold increase of *k*_cat_/*K*_M_). This is in agreement with previous reports on the increased peroxidase activity of the H64D mutant in comparison to wild‐type Mb.^[14]^

When FeCe6 was reconstituted into the swMb‐H64D protein scaffold, the catalytic efficiency of guaiacol oxidation increased 2.2 times and approximately 19 times compared to swMb‐H64D and FeCe6‐Mb, respectively, which arose from improved affinity to the substrate and, in the case of FeCe6‐Mb‐H64D *vs*. FeCe6‐Mb, from increased *k*_cat_. In TCP dehalogenation, the catalytic efficiency of FeCe6‐Mb‐H64D was 3.6 times higher than the catalytic efficiency of FeCe6‐Mb but around 25 times lower than the catalytic efficiency of swMb‐H64D.

MnCe6‐swMb‐H64D showed the highest substrate affinity to guaiacol among all the Mb variants and mutants, but substrate inhibition restricted its *k*_cat_ to 0.06 s^−1^. As a result, the catalytic efficiency of MnCe6‐swMb‐H64D in guaiacol oxidation was 6 times higher than MnCe6‐Mb and 3.5 times lower than swMb‐H64D. There was no conversion when MnCe6‐swMb‐H64D was applied to catalyse the dehalogenation reactions of TCP.

These results show that replacement of the distal H64 improved the H_2_O_2_ binding and activation of swMb‐H64D and FeCe6‐swMb‐H64D. The artificial cofactor Fe‐Ce6 improved substrate affinity of both FeCe6‐Mb and FeCe6‐swMb‐H64D compared to the PPIX variants. These additive effects helped Mb variants containing Fe‐Ce6 to exhibit higher catalytic efficiency in guaiacol oxidations. However, Fe‐Ce6 did not enhance *k_cat_* on both substrates, which limited the catalytic efficiency of Fe‐Ce6 containing Mb. Another interesting observation was that the microenvironment created by the distal Asp residue was apparently not compatible with Mn‐Ce6 given the poor performance of MnCe6‐swMb‐H64D on both substrates. The decreased activity of MnCe6‐swMb‐H64D on guaiacol and the ‘lost’ activity on TCP substrate can be taken as an indicator that the Mn=O intermediate of MnCe6‐swMb‐H64D may not be stable enough to catalyse the oxidation of both substrates. As hypothesised earlier, TCP must bind properly inside the active cavity to complete the reaction, while guaiacol is able to ‘catch’ electrons from the protein surface or from the edge of cofactor. Thus, the unstable Mn=O of MnCe6‐swMb‐H64D failed to convert TCP.^[34]^

## Conclusion

In summary, twelve Mb variants containing artificial cofactors based on six metal ions and two macrocycles were prepared. Their peroxidase activity on the oxidation of guaiacol and the dehalogenation of TCP was compared. While some artificial cofactor Mb variants have been characterized previously,^[35]^ to our knowledge a systematic comparison of these variants under identical reaction conditions has not been performed. Moreover, except for FeCe6‐Mb,^[19e]^ the other Ce6‐containing Mb variants were reconstituted and characterized for the first time. Under optimised reaction conditions, five Mb variants containing three types of metal ions had detectable peroxidase activities, showing that the type of metal ion and the porphyrin ligand both have impact on peroxidase activity. A more diverse series of metal centres led to active peroxidases when Ce6‐based cofactors were reconstituted in Mb. For instance, CuPPIX‐Mb was not able to catalyse the oxidation of guaiacol or TCP, while CuCe6‐Mb was active on both substrates. MnPPIX‐Mb could only catalyse the TCP dehalogenation, while MnCe6‐Mb was able to oxidize both substrates. Compared to FePPIX‐Mb, FeCe6‐Mb and MnCe6‐Mb were identified as variants with improved catalytic efficiency in guaiacol oxidation and TCP dehalogenation. These two variants showed increased substrate affinity, especially towards guaiacol. However, the *k*_cat_ of four active variants were lower than FePPIX‐Mb. Given the structural differences between the two set of cofactors, it is theorised that the electron deficient nature of Ce6 may result in the distortion of its structure and alter the p*K*_a_. This results in unfavourable conditions for H_2_O_2_ activation and Mb active species formation. There are ongoing investigations with regards to the decreased *K*_M_, and future experimental work will focus on the determination of redox potentials and elucidation of the structural properties of Ce6 variants.

## Experimental Section

A detailed experimental section can be found in the Supporting Information, the most important experiments are described in shorter form here. *In vitro* reconstitution of artificial myoglobin with cofactors was carried out in two steps. Firstly, the removal of the iron‐protoporphyrin IX (Fe‐PPIX, heme) was based on the Teale's method: The cofactor was extracted from the protein via cold 2‐butanone in acidic conditions (pH 2.5) followed by extensive dialysis to refold the protein (apo‐Mb). Second, a cold solution of cofactor in dimethylformamide (DMF) was added to the apo‐Mb solution while slowly shaking at 4 °C. The molar ratio between the cofactor and apo‐Mb was close to 5 : 1, while keeping the organic solvents lower than 2 % v/v. The mixture was then dialyzed against a 100‐fold volume of desired buffer and purified via a Sephadex G‐25 column to remove free cofactor. Final protein solution was concentrated with Amicon ultra‐centrifugal filter device to a desired concentration.

UV‐Vis spectroscopy was used to measure the Soret peak absorbance of Mb samples. For ICP‐MS, the concentration of metal species was kept lower than 500 μg L^−1^. Negative control was carried out by measuring ion concentrations in 2 % nitric solution, positive control was conducted by measuring the commercially available Mb in 2 % nitric solutions. The molar extinction coefficient of the Mb variant was calculated based on the obtained concentration of metal ion from ICP‐MS and the original sample's UV‐Vis absorbance of the Soret peak.

For CD spectroscopy, the concentration of protein samples was firstly measured (Lowry method) and then adjusted to 0.1 μg mL^−1^. Samples were measured in 2 mm pathlength quartz cuvettes. The sample scan was from 260 to 185 nm with 1 nm resolution, 1 nm bandwidth, 100 mdeg sensitivity and 50 nm min^−1^ scan speed. Spectra were averaged from three consecutive scans and smoothed over five data points. The measurement was carried out at 22 °C in 20 mM Tris‐HCl buffer (pH 8.0). The spectra were smoothed by the means‐movement method using Jasco Spectra Analysis software and subjected to secondary structure analysis using CDSSTR provided by DICHROWEB.^[36]^

*E. coli* BL21(DE3) cells were plated on a lysogeny broth (LB) agar plate containing 50 μg mL^−1^ kanamycin. A single colony of freshly transformed cells was cultured overnight in 3 mL of LB medium containing 50 μg mL^−1^ kanamycin. Then, 1 mL of the culture was used to inoculate 100 mL of Terrific‐Broth (TB) medium supplemented with 50 μg mL^−1^ kanamycin. The culture was incubated for ∼3 h at 37 °C at a shaking speed of 230 r.p.m. When the absorbance of the culture at a wavelength of 600 nm reached 1.0, 0.1 mM isopropyl β‐d‐1‐thiogalactopyranoside (IPTG) and 0.3 mM δ‐aminolaevulinic acid (1 M stock) were added to induce expression of the Mb protein. The induced cultures were incubated for ∼18 h at 22 °C, and the cells were subsequently harvested by centrifugation at 4000 g for 20 min. The pelleted bacterial cells were suspended in 50 mL phosphate buffered saline (PBS) buffer (Na_2_HPO_4_ 10 mM, KH_2_PO_4_ 1.8 mM, NaCl 137 mM, KCl 2.7 mM, pH 7.4) and disrupted by sonication.

The peroxidase activity assay of Mb and its variants in PBS buffer (pH 7.4) was performed by UV‐Vis spectroscopy with a dual mixing stopped‐flow unit (Applied Photophysics RX200) at 22 °C. The absorbance at 272 nm (12 mM^−1^ cm^−1^) of TCP's oxidation product was monitored versus time. The absorbance at 470 nm (26.6 mM^−1^ cm^−1^) of guaiacol's oxidation product was monitored versus time. The initial rate (*V*_0_) for each reaction was calculated from the initial liner part of the absorbance trace. To determine the enzymatic steady state kinetic (*k*_cat_ and *K*_M_), varied concentrations of substrate (guaiacol or TCP) were mixed with enzyme sample in syringe A and the H_2_O_2_ was in syringe B. The measurement was initiated by mixing same volumes from the two syringes. The plots of initial rates as a function of substrate concentration were fitted to the Michaelis‐Menten equation. Nonlinear curve fit and analysis was performed in OriginPro 2020 with Enzyme Kinetics application.

## Conflict of interest

The authors declare no conflict of interest.

1

## Supporting information

As a service to our authors and readers, this journal provides supporting information supplied by the authors. Such materials are peer reviewed and may be re‐organized for online delivery, but are not copy‐edited or typeset. Technical support issues arising from supporting information (other than missing files) should be addressed to the authors.

Supporting Information

## Data Availability

The data that support the findings of this study will be made openly available upon acceptance of the article and before its final publication in the repository of the University of Strathclyde https://pureportal.strath.ac.uk/en/datasets/.
