# Supplementary material for: Peroxidase Activity of Myoglobin Variants Reconstituted with Artificial Cofactors
Source: Chembiochem. 2022 Jul 28;23(18):e202200197. doi: 10.1002/cbic.202200197 (PMC9545363; doi:10.1002/cbic.202200197)
Supplement: Supplementary file 1 — Supporting Information [file CBIC-23-0-s001.pdf]

ChemBioChem

Supporting Information

Peroxidase Activity of Myoglobin Variants Reconstituted with Artificial Cofactors

Chao Guo[+], Robert J. Chadwick[+], Adam Foulis, Giada Bedendi, Andriy Lubskyy, Kyle J. Rodriguez, Michela M. Pellizzoni, Ross D. Milton, Rebecca Beveridge, and Nico Bruns*

Table of Contents

1. Experimental Details 1

1.1 Materials and Instruments 1

1.2 Mb Mutagenesis Protocol 2

1.3 Protocol for Plasmid Transformation 3

1.4 Protocol for Mb Expression 3

1.5 Protocol for Native Mb Purification 4

1.6 Synthesis of Cofactors 5

1.7 Reconstitution of Mb Variants 6

1.8 Characterisation of Protein Samples by UV-Vis Spectroscopy 6

1.9 Characterisation of Mb Variants by CD Spectroscopy 7

1.10 Sample Preparation for ICP-MS Analysis 7

1.11 Characterisation of Mb Variants by SDS-PAGE 8

1.12 Characterisation of Mb Variants by Native Mass Spectrometry 8

1.13 Spectroelectrochemical Redox Potential Determination 9

1.14 Stopped-flow Colorimetric Peroxidase Assays 9

1.15 Stopped-flow Spectroscopy of Mb Variants in the Presence of H_2O_2 10

2. Supporting Figures and Tables 11

3. References 28

1. Experimental Details

1.1 Materials and Instruments

The commercial Mb was purchased from Sigma-Aldrich, the *E.coli* Top 10 and BL21 (DE3) cells and the plasmid pET-30(a)-eYFP was a gift from the Clark lab (Department of Chemical Engineering, University of California, Berkeley)[1], the sperm whale myoglobin (swMb) gene was synthesised by GenScript with codon optimization. High-fidelity polymerase (Phusion) was purchased from Thermo Fisher, the T4 ligase was purchased from New England Biolabs. The yeast extraction and tryptone for culture was from BD Biosciences. Isopropyl β-D-1-thiogalactopyranoside (IPTG), δ-aminolevulinic acid (5-ALA) and kanamycin were ordered from Sigma-Aldrich. Other chemicals and solvents used were used as received from commercial supplier without further purification. Lab ultrapure water was from a Milli-Q integral 15 water purification system. All expression media were sterilized by either autoclave (45 min, 121 °C, Astell AMB440) or a sterile syringe filter (0.22 µm). To maintain sterile conditions, all the related experiments were manipulated in a class II biosafety cabinet (Thermo Scientific S2020 1.2).

PCR reactions were performed with a SimpliAmp thermal cycler (Applied Biosystems), *E.coli* Top10 and BL21 (DE3) competent cells were prepared following the Hanahan method[2], cell lysis was carried out by sonication (120 W, 20 kHz, Fisherbrand Model 120 Sonic Dismembrator, Fisher Scientific), UV-Vis spectra were recorded on Cary 60 (Agilent Technologies), CD spectra were recorded on Jasco-J810 spectropolarimeter using quartz cuvettes. Inductively Coupled Plasma Mass Spectrometry (ICP-MS) was performed on an Agilent 7700 instrument. Protein purification was carried out on ÄKTA pure chromatography system equipped with columns from GE Healthcare. Sodium dodecyl sulphate-polyacrylamide gel electrophoresis (SDS-PAGE) was run on a Mini-PROTEAN Tetra Vertical Electrophoresis Cell (Bio-Rad).

Sodium Phosphate (NaPi) and PBS buffer was prepared based on the recipe from Cold Spring Harbor protocols.[3] PBS-Br buffer was modified from common PBS buffer as 100 mM NaBr, 10 mM Na_2HPO_4, 2 mM KH_2PO_4.

1.2 Mb Mutagenesis Protocol

The primers were designed according to guidelines (Agilent QuickChange II site-directed mutagenesis kit). Their melting temperature (T_m) were estimated with the formula:
T_m = 81.5 + 0.41(%GC) - (675/N) - %mismatch,
where N is the primer length in bases. Ideal T_m should be higher than 78 °C.
The length of primers was controlled between 25 to 45 bases. All the primers were synthesised by integrated DNA Technologies. The mutant strand synthesis was completed by thermal cycling reactions based on the indicated conditions below:

Reaction reagents	
1 μL	plasmid template (100 ng μL^{-1})
1.25 μL	Fw-primer (100 ng μL^{-1})
1.25 μL	Rv-primer (100 ng μL^{-1})
2 μL	dNTPs (25 mM each)
10 μL	5X buffer
1 μL	Phusion II
33.5 μL	ddH_2O
PCR parameters (30 cycles)	
98 °C	30 s
98 °C	10 s
55 °C	60 s
72 °C	3 m
72 °C	10 m

The PCR products were digested by Dpn I at 37 °C for an hour before the transformation was carried out.

The Mb H64D mutant was prepared with the use of the following primers:

H64D-Fw	5'-GAT CTG AAG AAA **GAT** GGT GTG ACC GTT CTG ACC-3'
H64D-Rv	5'-CAG GGT CAC ACC **ATC** TTT CTT CAG ATC TTC GCT- 3'

Coding DNA Sequence of swMb

ATGGTGCTGAGCGAGGGTGAATGGCAGCTGGTGCTGCACGTTTGGGCGAAAGTGGAGGCGGA
CGTTGCGGGTCACGGCCAAGATATCCTGATTCGTCTGTTCAAAAGCCACCCGGAAACCCTGG
AAAAGTTCGACCGTTTTAAACACCTGAAGACCGAGGCGGAAATGAAGGCGAGCGAAGATCTG
AAGAAACATGGTGTGACCGTTCTGACCGCGCTGGGTGCGATCCTGAAGAAAAAGGGCCACCA
CGAGGCGGAACTGAAACCGCTGGCGCAGAGCCACGCGACCAAACACAAGATCCCGATTAAGT
ACCTGGAGTTTATTAGCGAAGCGATCATTCACGTTCTGCATAGCCGTCATCCGGGTGACTTT
GGTGCGGATGCGCAGGGTGCGATGAACAAAGCGCTGGAGCTGTTTCGTAAGGACATCGCGGC
GAAATACAAGGAACTGGGTTATCAAGGC

1.3 Protocol for Plasmid Transformation

PCR digestion products or complete plasmid were added into the competent cell solution. The mixture was hold on ice for half hour and then heated at 42 °C for around 45 seconds. 7x volume of Luria-Bertani (LB) medium was added into the mixture and kept shaking one hour at 37 °C at a shaking speed of 230 r.p.m. 100 μL of the mixture was loaded on LB agar plate containing the corresponding antibiotics. The plate was kept in incubator at 37 °C for overnight.

1.4 Protocol for Mb Expression

Escherichia coli BL21(DE3) cells were plated on a lysogeny broth agar plate containing 50 μg•ml^{-1} kanamycin. A single colony of freshly transformed cells was cultured overnight in 3 ml of LB medium containing 50 μg•ml^{-1} kanamycin. Then, 1 ml of the culture was used to inoculate 100 ml of Terrific-Broth (TB) medium supplemented with 50 μg•ml^{-1} kanamycin. The culture was incubated for ~3 h at 37 °C at a shaking speed of 230 r.p.m. When the absorbance of the culture at a wavelength of 600 nm reached 1.0, 0.1 mM IPTG and 0.3 mM δ-aminolaevulinic acid (1 M stock) were added to induce expression of the Mb protein. The induced cultures were incubated for ~18 h at 22 °C, and the cells were subsequently harvested by centrifugation at 4,000 g for 20 min. The pelleted bacterial cells were suspended in 50 mL phosphate buffered saline (PBS) buffer (Na_2HPO_4 10 mM, KH_2PO_4 1.8 mM, NaCl 137 mM, KCl 2.7 mM, pH 7.4) and disrupted by sonication (Fisherbrand Model 120 Sonic

Dismembrator). Sonication Program: 2 seconds working/2 seconds pulse, 10 mins working time totally.

1.5 Protocol for Native Mb Purification

To maximize heme occupancy, swMb was reconstituted with heme chloride. Briefly, the lysate solutions were rapidly mixed with 0.2 mM of heme chloride (20 mM stock solution in 10 mM NaOH) and incubated at 4 °C for 10 min. The lysate was centrifuged at 7500 g for 20 min, the supernatant was transferred to another Falcon tube and the same centrifuge was applied again. After this, the supernatant was filtered through a 0.25 μm pore size membrane filter before being subjected to Ni-NTA agarose chromatography.

After 4 column volumes (CV) equilibration with PBS buffer, the column was washed with 6 CV of buffer (50 mM NaPi, 250 mM NaCl, 10 mM imidazole, pH 8.0), the proteins were eluted with elution buffer (50 mM NaPi, 250 mM NaCl, 250 mM imidazole, pH 8.0). Elution was in block phases, 2 CV from 0% elution buffer to 10%, and then keeping 10% elution buffer for 2 CV, increasing the concentration of elution buffer to 100% with 5 CV and keeping this elution condition until target protein was collected.

The protein solutions were concentrated to 1 mL in PBS using Amicon Ultra-15 (10 kDa cut-off) centrifugal filter device (Merck Millipore), and then subjected to size-exclusion chromatography on 16/60 SF300 (GE Healthcare Life Sciences) equilibrated with PBS at a flow rate of 0.8 ml•min^{-1}. The fractions were monitored at 280 and 410 nm, and samples collected using a fraction collector.

The protein solution was concentrated with an Amicon Ultra-15 (10 kDa cut-off) centrifugal filter device (Merck Millipore). The concentration of native Mb was determined by UV–Vis absorbance measurements at 407 nm using an extinction coefficient 188 mM^{-1}•cm^{-1}.[4]

1.6 Synthesis of Cofactors

A) B)

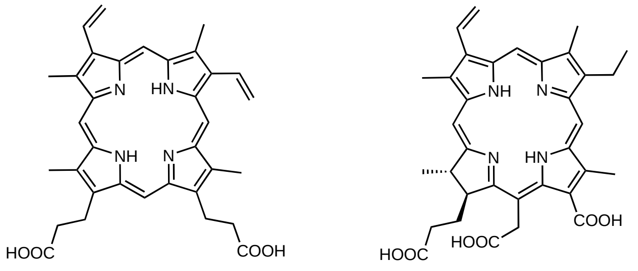

protoporphyrin IX chlorin e6

Scheme S1. Structures of protoporphyrin IX and chlorine e6.

Fe(III)-PPIX (CAS 16009-13-5), Cu(III)-PPIX (CAS 14494-37-2), Co(III)-PPIX (CAS 102601-60-5), Ni(II)-PPIX (CAS 15415-30-2), and Mn(III)-PPIX (CAS 120389-54-0) were purchased from Frontier Scientific and used as received.

Chlorin e6 (10 mg) was dissolved in acetone (10 mL) firstly and $FeCl_2 \cdot 4\ H_2O$ (12 mg) was added to a solution (+)-L-ascorbic acid (36 mg) in acetone (10 mL). Two solutions were mixed to start the reaction. At 60 °C, the reaction mixture was stirred for 6 hours. 10 mL dichloromethane were added and the mixture rinsed with saturated brine solution (3 x 20 mL), then with 10 mL of 0.01 M HCl solution, and finally with water after cooling to room temperature (20 mL). Fe(III)-Ce6 was obtained as a dark green powder when the organic layer was dried and evaporated. All the procedures were carried out in complete darkness under argon atmosphere. LC -MS: m/z ($[M – Cl]^+$) calc. 650.2; obs. 650.0.

Cu-Ce6/Co-Ce6/Mn-Ce6/Ni-Ce6/Ru-Ce6/Ru-PPIX were synthesised with the reaction conditions stated in the following Table.

Reaction conditions of cofactor synthesis

Metal (mass)	Cofactor (mass)	Solvent	Reaction Temp. (°C)	Reaction Time (h)
$Cu(CH_3COO)_2$ (20 mg)	Ce6 (10 mg)	MeOH	80 (reflux)	4
$Co(CH_3COO)_2$ (20 mg)	Ce6 (10 mg)	MeOH	80 (reflux)	4
$Mn(CH_3COO)_2$ (20 mg)	Ce6 (10 mg)	MeOH	80 (reflux)	5
$NiCl_2$ (20 mg)	Ce6 (10 mg)	MeOH	80 (reflux)	5
$Ru_3[(CO)_3]_4$ (20 mg)	Ce6 (10 mg)	DMF	120 (reflux)	24
$Ru_3[(CO)_3]_4$ (20 mg)	PPIX (10 mg)	DMF	120 (reflux)	24

Under positive argon pressure, the reagents were dissolved in specific solvents and heated under reflux at the mentioned temperatures. After this, the solvent was removed under reduced pressure, the residue taken up in a small amount of MeOH, filtered and re-dissolved in CH_2Cl_2. The crude product was purified by silica column. Unreacted salt was first eluted with CH_2Cl_2, followed by unreacted free base. The cofactor product was collected by elution

with 1:1 MeOH and CH_2Cl_2 mixture. After the solvent was removed under reduced pressure, the product was re-dissolved in DMF and filtered with a 0.45 μm pore size membrane filter for further use. Synthesised cofactors were characterised by UV-Vis spectroscopy and LC-MS. It should be noted that the workup and storage of the cofactors was carried out in air, which leads to oxidation of the complexes to their most stable form.

LC-MS results of synthesised cofactors

Cofactor	Cu(III)-Ce6	Co(III)-Ce6	Mn(III)-Ce6	Ni(II)-Ce6	Ru(II)-Ce6	Ru(II)-PPIX
calc. mass m/z	657.15 $(M-Cl)^+$	653.15 $(M-Cl)^+$	649.15 $(M-Cl)^+$	652.15 $(M-Cl)^+$	724.15 $(M+CO)^+$	677.17 $(M-CO)^+$
obs. mass m/z	657.3	654.3	652.4	654.2	724.4	678.6

1.7 Reconstitution of Mb Variants

In vitro reconstitution of artificial myoglobin with non-native cofactors was carried out in two steps. Firstly, the removal of the iron-protoporphyrin IX (Fe-PPIX, heme) was based on the Teale's method [5]: extraction of the cofactor via cold 2-butanone in acidic condition (pH 2.5) and followed by extensive dialysis against PBS buffer to refold the protein (apo-Mb). Second, an equal volume of cold solution of excess (10 eqiv.) cofactors in dimethylformamide (DMF) was added to 1 mL of the apo-Mb solution with slowly shaking at 4 °C. The molar ratio between the cofactor and apo-Mb was close to 5:1, while keeping the organic solvents lower than 2% v/v. The mixture was then dialyzed against a 100-fold volume of desired buffer (via 10 KDa cut-off dialysis tube) and purified via a Sephadex G-25 column to remove free cofactor. Final protein solution was concentrated with an Amicon ultra centrifugal filter device to desired concentration. The myoglobin variants were maintained at 4 °C and used within 2 days.

1.8 Characterisation of Protein Samples by UV-Vis Spectroscopy

Before the sample measurement, the baseline was corrected with the blank buffer. The scan rate was set to 4800 nm min^{-1} with 1 nm interval. All the protein samples without specific mentions were measured in PBS buffer at 22 °C. Cofactor characterisation was measured in Milli-Q water containing 1% DMF. Soret peak, Q bands and A280 were collected to characterise Mb samples.

1.9 Characterisation of Mb Variants by CD Spectroscopy

The concentration of protein samples was firstly measured (Lowry method) and then adjusted to 0.1 μg•mL^{-1}. Samples were measured in 2 mm pathlength quartz cuvettes. The sample scan was from 260 to 185 nm with 1 nm resolution, 1 nm bandwidth, 100 mdeg sensitivity and 50 nm•min^{-1} scan speed. Spectra were averaged from three consecutive scans and smoothed over five data points. The measurement was carried out at 22 °C in 20 mM Tris-HCl buffer (pH 8.0). The spectra were smoothed by the means-movement method using Jasco Spectra Analysis software and subjected to secondary structure analysis using the analysis programme CDSSTR (Reference set 6) provided by DICHROWEB.[6] Reference set 6 was selected because of its match with the wavelength range. It also contains myoglobin, and heamoglobin.

1.10 Sample Preparation for ICP-MS Analysis

Protein samples were firstly desalted with Milli-Q water using Amicon centrifugal filter device. UV-Vis spectroscopy was used to measure the Soret peak absorbance and to estimate the metal concentration. The protein samples were then diluted with 2% nitric acid solution. The concentration of metal specie was kept lower than 500 μg•L^{-1} and then ICP-MS was measured. The molar extinction coefficient of Mb variant was calculated based on the obtained concentration of metal ion from ICP-MS and the original sample's UV-Vis absorbance on the Soret peak.

1.11 Characterisation of Mb Variants by SDS-PAGE

Separation gel (10%) and stacking gel were prepared based on the following order:

Reagent	separation gel	stacking gel
H_2O	4.1 mL	6.1 mL
Acrylamide/bis (30% 37.5:1; Bio-Rad)	3.3 mL	1.3 mL
Tris–HCl (1.5 M, pH 8.8, separating gel) (0.5 M, pH 6.8, stacking gel)	2.5 mL	2.5 mL
SDS, 10%	100 µL	100 µL
N,N,N′,N′-tetramethylethylene-diamine (TEMED)	10 µL	10 µL
Ammonium persulfate (APS), 10%	32 µL	100 µL

Protein samples were mixed with loading buffer (0.125 m Tris-Cl pH 6.8, 0.5% SDS, 10% (v/v) glycerol, 0.001% bromophenol blue) and heated at 95 °C for 5 minutes. Samples were then centrifuged to remove sediments and loaded into gel lanes starting with molecular ladder solution. The SDS-PAGE experiment was performed in SDS-PAGE running buffer (25 mM Tris, 192 mM glycine and 0.1% SDS, pH 8.3) at 120 V for at least 120 minutes. Gels were stained with standard Coomassie stain protocol.[7]

1.12 Characterisation of Mb Variants by Native Mass Spectrometry

Protein samples were desalted and buffer exchanged into 100 mM ammonium acetate with 10 kDa filters and diluted to an approximate protein concentration of 5 µM. Mass spectrometry experiments were carried out on a Waters Synapt G2-Si mass spectrometer (Waters Corporation, Manchester, UK) using a nano electrospray ionisation source (nESI). Tips for nESI were pulled in house from thin-walled borosilicate glass capillaries (i.d. 0.78 mm, o.d. 1.0 mm) (Sutter Instrument Co., Novato, CA, USA) using a Flaming/Brown micropipette puller (Sutter Instrument Co., Novato, CA, USA). A positive potential was applied to the analyte solution via a thin platinum wire (diameter 0.125 mm) (Goodfellow, Huntingdon, UK). Mass calibration was performed by a separate infusion of sodium iodide (NaI) cluster ions. The following instrument parameters were used: capillary voltage 0.9-1.6 kV, sample cone voltage 30-100 V, source offset 40-120 V, source temperature 40 °C, trap collision energy 4.0 V, trap

gas 2-3 mL min^{-1}. Mass spectrometry data was processed using MassLynx and mass spectra figures were produced using Unidec.

1.13 Spectroelectrochemical Redox Potential Determination

The redox potentials of FePPIX-Mb and FeCe6-Mb were determined using an Autolab UV/VIS/NIR spectrophotometer coupled to an Autolab PGSTAT204 (Metrohm AG, Switzerland) (Figure S7). The quartz spectroelectrochemical cell (10 mm pathlength) was housed within an Ar-filled anoxic glovebox (Jacomex, France, <0.5 ppm O_2) and connected to the external instrumentation using 200 µm fiber optic cables. All experiments were performed at 25 ºC using a Peltier temperature-controlled, stirred cuvette holder. A Pt grid electrode was used as the working electrode alongside an Ag/AgCl (3 M KCl) reference electrode, and a coiled Pt counter electrode was shielded from the main electrochemical cell behind a CoralPor® glass tip. The reference electrode was monitored daily against a pristine saturated calomel electrode (SCE, saturated KCl), and all redox potentials were subsequently reported *versus* the standard hydrogen electrode (SHE) by the following relationship: $E_{SHE} = E_{SCE} + 0.242$ V. The redox potential of the electrochemical cell (typically containing 3.5 µM Mb alongside 10 µM methylene blue) was stepped with 50 mV intervals between 0 to −0.3 V vs. Ag/AgCl (3 M KCl), where each step was applied for 120 s to ensure equilibration of the cell. All recorded absorbances were corrected against control experiments containing only methylene blue. Corrected absorbance values were subsequently determined by averaging the final 30 s of each step. Data were fitted to the Nernst equation by nonlinear regression using GraphPad Prism. All experiments were performed in triplicate (error = standard deviation).

1.14 Stopped-flow Colorimetric Peroxidase Assays

The peroxidase activity assay of Mb and its variants in PBS buffer (pH 7.4) was performed on a UV-Vis spectrometer equipped with a dual mixing stopped-flow unit (Applied Photophysics RX200) at 22 °C. The absorbance at 272 nm (12 mM^{-1}•cm^{-1}) of TCP's oxidation product was monitored *versus* time. The absorbance at 470 nm (26.6 mM^{-1}•cm^{-1}) of guaiacol's oxidation product was monitored *versus* time. To screen the reactivity of FePPIX-Mb and other variants toward guaiacol and TCP, the reaction condition was based on the Table S5. Kinetic parameters were determined using the concentrations reported in Table S6. The concentration of each Mb variant, as detailed in Table S5 and S6, was selected to allow for good colorimetric signal development within the 0.5 min of each kinetic measurement. The initial rate (V_0) for each reaction was calculated from the liner initial part of the absorbance

trace. To determine the enzymatic steady state kinetic (k_{cat} and K_m), varied concentration of substrate (guaiacol or TCP) was mixed with enzyme sample in syringe A (Table S6) and the H_2O_2 was in syringe B.

The measurement was initiated by mixing of two syringes in same volumes. The plots of initial rates as a function of substrate concentrations were fitted to the Michaelis-Menten equation (Equation 1.1). Nonlinear curve fit and analysis was performed in OriginPro 2020 with Enzyme Kinetics application.

$$\frac{V_0}{[enzyme]} = \frac{k_{cat}[substrate]}{K_m + [substrate]}$$ **Equation 1.1**

1.15 Stopped-flow Spectroscopy of Mb Variants in the Presence of H_2O_2

Kinetic studies of FePPIX-Mb and Mb variants (FeCe6-Mb, CuCe6-Mb, MnCe6-Mb and MnPPIX-Mb) in reaction with H_2O_2 were performed with the same stopped-flow spectroscopy method as described above. Generally, one syringe contained 20 μM protein, and the second syringe contained various concentrations of H_2O_2 (0.2 -1.6 mM). The reaction was stated with mixing of equal volume of solutions from both syringes. Over the course of one minute, the absorbances of Soret peak were recorded at every 0.05 second (Table 9.4). The decay of the Soret peak were plotted, and the observed rate constants (k_{obs}, 1/t, s^{-1}) were calculated from single-exponential fits (Equation 1.2). The apparent rate constants (k_{app}, $mM^{-1} \cdot s^{-1}$) for compound II formation were obtained by linear regression fitting the plot of the observed rate constants, k_{obs}, versus the concentrations of H_2O_2 (Equation 1.3).[8]

$$y = Ae^{-\frac{x}{t}} + y_0$$ **Equation 1.2**

$$k_{obs} = k_{off} + k_{app}[H_2O_2]$$ **Equation 1.3**

Here, k_{off} represents the dissociation rate constant of the Mb-H_2O_2 complex.

2. Supporting Figures and Tables

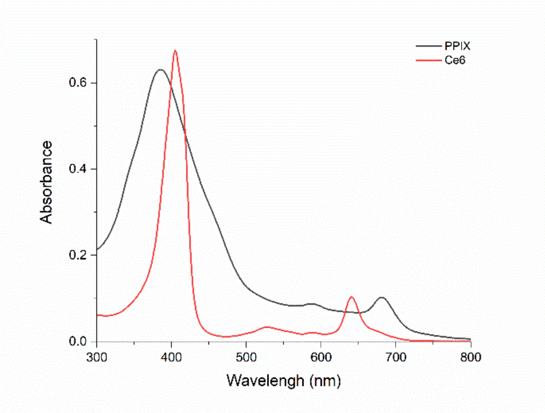

Figure S1. UV-Vis spectra of free PPIX and Ce6 in the absence of metal ions.

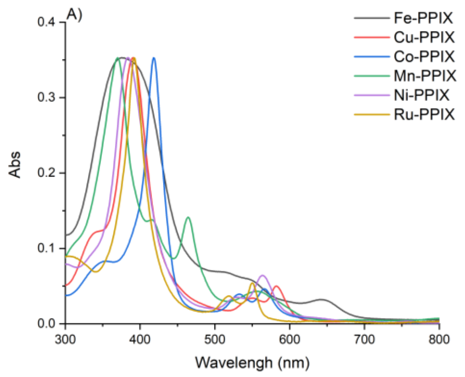

Figure S2. UV-Vis spectra of iron(III), copper(III), cobalt(III), manganese(III), nickel(II) and ruthenium(II) PPIXs complexes.

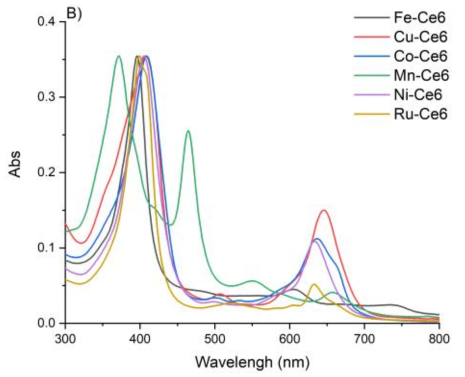

Figure S3. UV-Vis spectra of iron(III), copper(III), cobalt(III), manganese(III), nickel(II) and ruthenium(II) Ce6 complexes.

Table S1. UV-Vis absorbance bands of protoporphyrin IX, chlorin e6, and metal ion complexes of protoporphyrin IX and chlorin e6

Complex	Soret band (nm)		Q band (nm)	
			α	β
Ce6	405	-	641	527
Fe-Ce6	396	-	742	606
Cu-Ce6	408	-	645	508
Co-Ce6	409	-	637	502
Mn-Ce6	372	465	658	551
Ni-Ce6	405	-	634	500
Ru-Ce6	400	-	633	523
PPIX	385	-	681	588
Fe-PPIX	376	-	643	551
Cu-PPIX	391	-	583	550
Co-PPIX	418	-	566	532
Mn-PPIX	370	464	558	-
Ni-PPIX	384	-	528	564
Ru-PPIX	392	-	550	520

Table S2. UV-Vis absorbance bands of Mb variants

Enzyme	Soret band (nm)		Q band (nm)		Molar extinction coefficient (ε) of Soret band ($mM^{-1}\cdot cm^{-1}$) [a]	Reported ε ($mM^{-1}\cdot cm^{-1}$)
			α	β		
FePPIX-Mb	409	-	635	508	187	188[9]
CuPPIX-Mb	425	-	586	547	265	
CoPPIX-Mb	426	-	570	536	222	190[10]
MnPPIX-Mb	376	471	555	-	95	75[11]
NiPPIX-Mb	424		585	543	194	
RuPPIX-Mb	398	-	552	520	208	195[12]
FeCe6-Mb	407	-	640	-	110	188[13]
CuCe6-Mb	412	-	630	506	188	
CoCe6-Mb	427	-	642	-	104	
MnCe6-Mb	373	469	654	556	61	
NiCe6-Mb	408		628	-	158	
RuCe6-Mb	401	-	653	602	94	

[a] Mean value of duplicates are reported with respect to absorbances measured at the maximum wavelength of the Soret bands. (For MnPPIX-Mb and MnCe6-Mb, the absorbances were measured at 376 and 373 nm).

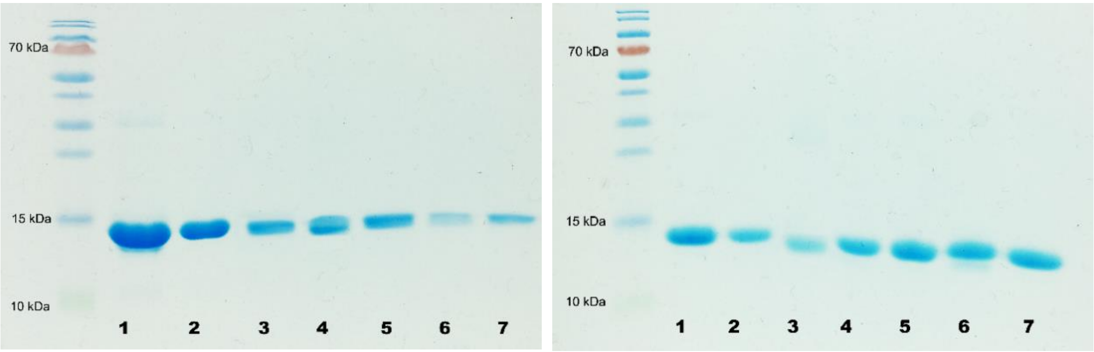

Figure S4. SDS-PAGE of Mb variants

Figure (left) shows the gel with Mb variants based on PPIX cofactors. From lane 1 to lane 7, the samples loaded were commercially available Mb, FePPIX-Mb, CuPPIX-Mb, CoPPIX-Mb, MnPPIX-Mb, NiPPIX-Mb and RuPPIX-Mb. Figure (right) shows the gel with Mb variants based on Ce6 cofactors. From lane 1 to lane 7, the samples loaded are commercially available Mb, FeCe6-Mb, CuCe6-Mb, CoCe6-Mb, MnCe6-Mb, NiCe6-Mb and RuCe6-Mb. The SDS-PAGE experiment was performed in SDS-PAGE running buffer (25 mM Tris, 192 mM glycine and 0.1% SDS, pH 8.3) at 120 V for 120 min. Gels were stained with Coomassie stain. Protein ladder was loaded into the first left lane, 10, 15 and 70 kDa were annotated in figures.

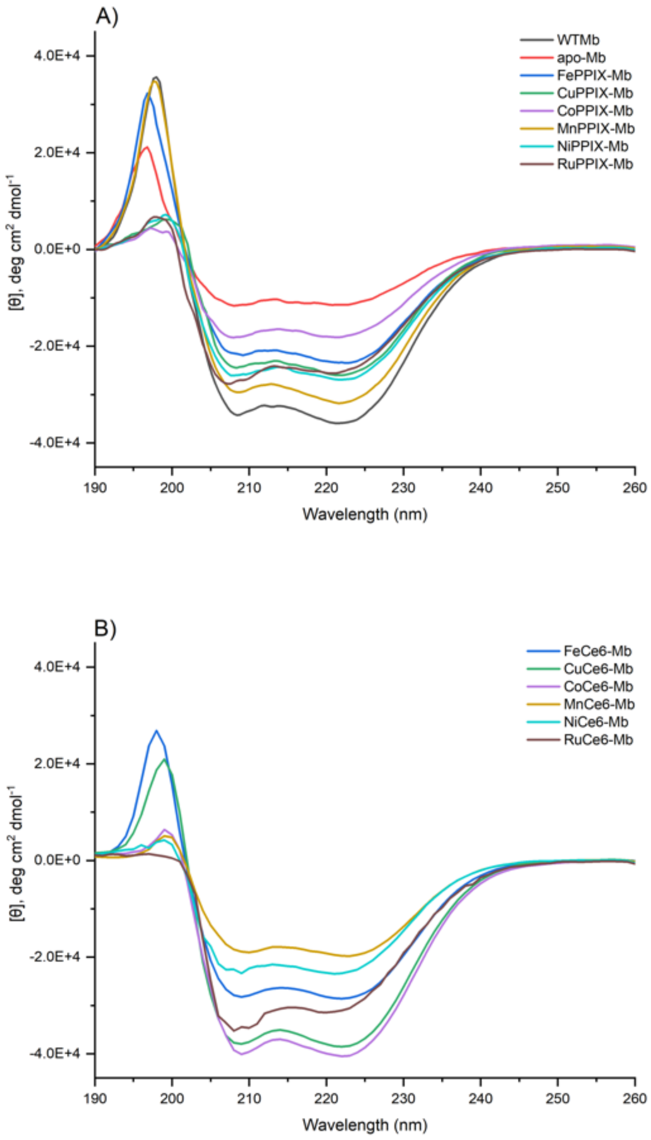
A)
WTMb
apo-Mb
FePPIX-Mb
CuPPIX-Mb
CoPPIX-Mb
MnPPIX-Mb
NiPPIX-Mb
RuPPIX-Mb
[θ], deg cm^2 dmol^{-1}
4.0E+4
2.0E+4
0.0E+0
-2.0E+4
-4.0E+4
190
200
210
220
230
240
250
260
Wavelength (nm)
B)
FeCe6-Mb
CuCe6-Mb
CoCe6-Mb
MnCe6-Mb
NiCe6-Mb
RuCe6-Mb
[θ], deg cm^2 dmol^{-1}
4.0E+4
2.0E+4
0.0E+0
-2.0E+4
-4.0E+4
190
200
210
220
230
240
250
260
Wavelength (nm)

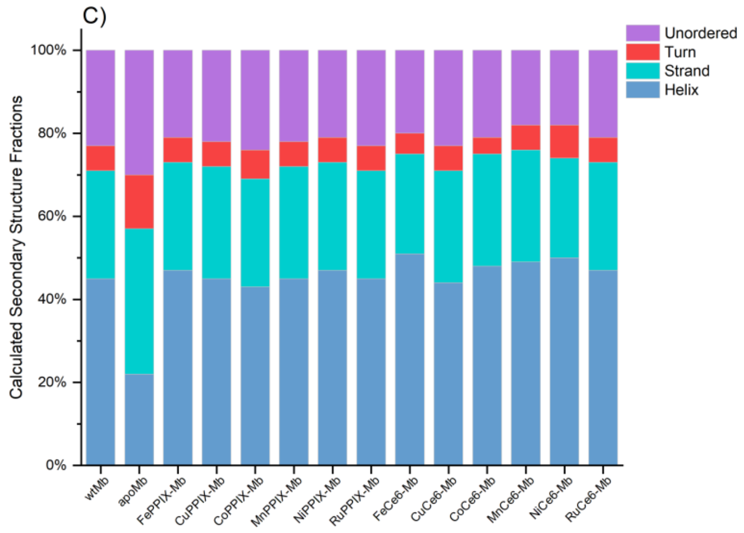

Figure S5. Circular dichroism spectra of Mb variants

Figure A) shows the overlayed CD spectra of Mb variants with PPIX cofactors and apoMb. B) shows the overlayed CD spectra of Mb variants with Ce6 cofactors. C) shows the calculated secondary structures of Mb variants. The spectra were smoothed by the means-movement method using Jasco Spectra Analysis software and subjected to secondary structure analysis using the analysis programme CDSSTR (Reference set 6) provided by DICHROWEB.[6] We note that the programme overestimates the presence of β-strand structures and have also tried other suitable reference sets, such as set 7, but did not find any significant differences. The measurement was carried out at 22 °C in 20 mM Tris-HCl buffer (pH 8.0). wtMb here refers to the commercially available Mb sample.

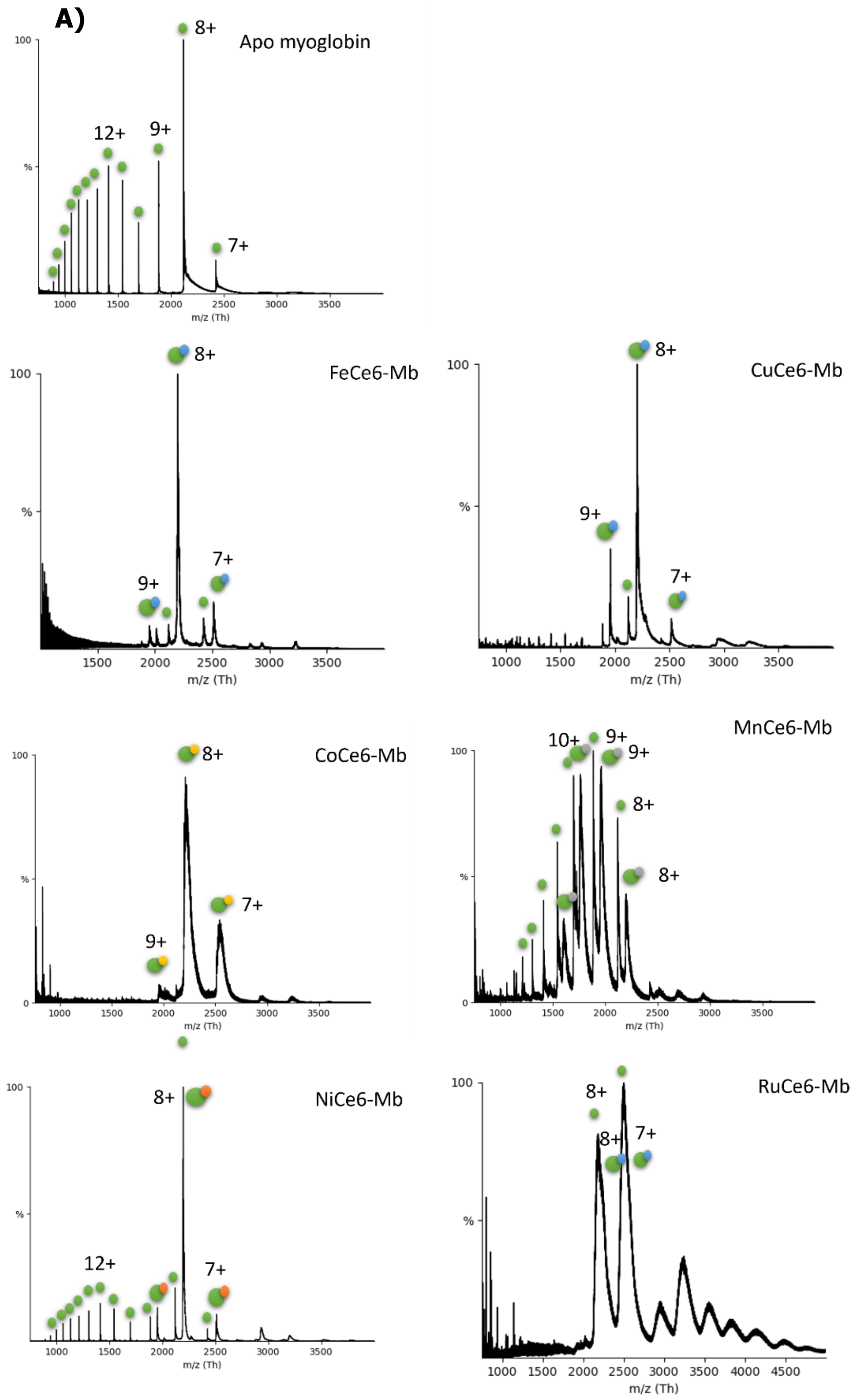
A)
Apo myoglobin
100
%
8+
12+
9+
7+
1000 1500 2000 2500 3000 3500
m/z (Th)
FeCe6-Mb
100
%
8+
9+
7+
1500 2000 2500 3000 3500
m/z (Th)
CuCe6-Mb
100
%
8+
9+
7+
1000 1500 2000 2500 3000 3500
m/z (Th)
CoCe6-Mb
100
%
0
8+
7+
9+
1000 1500 2000 2500 3000 3500
m/z (Th)
MnCe6-Mb
100
%
0
10+
9+
9+
8+
8+
1000 1500 2000 2500 3000 3500
m/z (Th)
NiCe6-Mb
100
%
8+
12+
7+
1000 1500 2000 2500 3000 3500
m/z (Th)
RuCe6-Mb
100
%
8+
8+
7+
1000 1500 2000 2500 3000 3500 4000 4500
m/z (Th)

B)

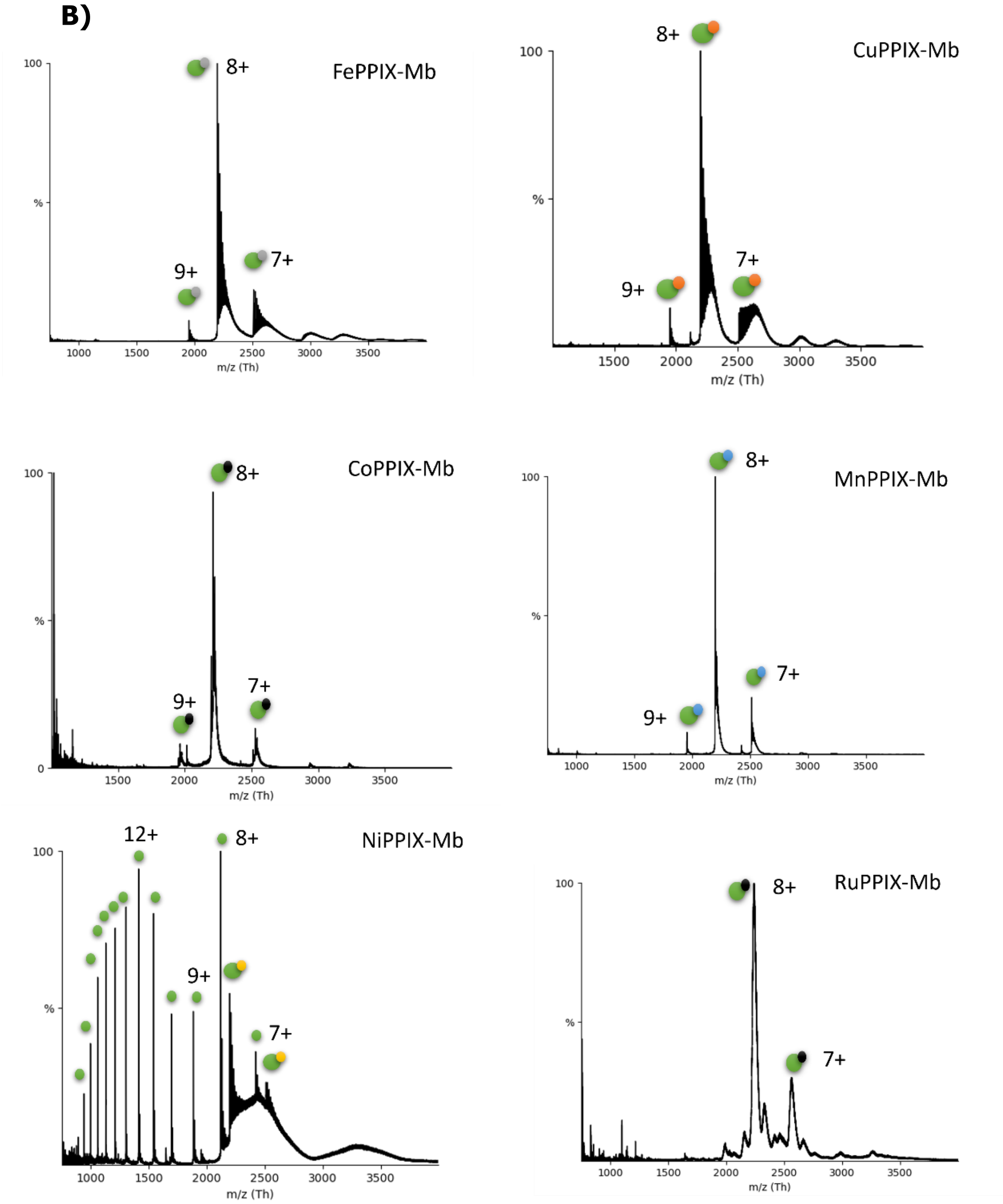

Figure S6. Native MS spectra of apo-Mb and Mb variants

Figure A) shows nMS spectra of apo-Mb and Mb variants with Ce6 cofactors. B) shows nMS spectra of Mb variants with PPIX cofactors. Charge states above 9+ correspond to unfolded myoglobin, with charge states 7+, 8+ and 9+ corresponding to compact conformations, suggesting that the cofactors stabilise the folded form of Mb.

Table S3: Native MS results of myoglobin with reconstituted cofactors including approximate molecular weight of the complex and whether the cofactor was completely or incompletely incorporated. Mass of complex is approximate due to unresolved salt and solvent adducts bound to the complex.

Cofactor	Approx. Mw of intact complex (Da)	Complete or partial cofactor incorporation
FeCe6	17 576	complete
CoPPIX	17 686	complete
MnPPIX	17 574	complete
CuPPIX	17 576	complete
FePPIX	17 566	complete
NiPPIX	17 578	partial
RUPPIX	17 672	complete
CoCe6	17 592	complete
NiCe6	17 526	partial
MnCe6	17 552	partial
CuCe6	17 612	Complete
RuCe6	17 928*	Partial

* very broad peaks difficult to resolve. Mw likely lower.

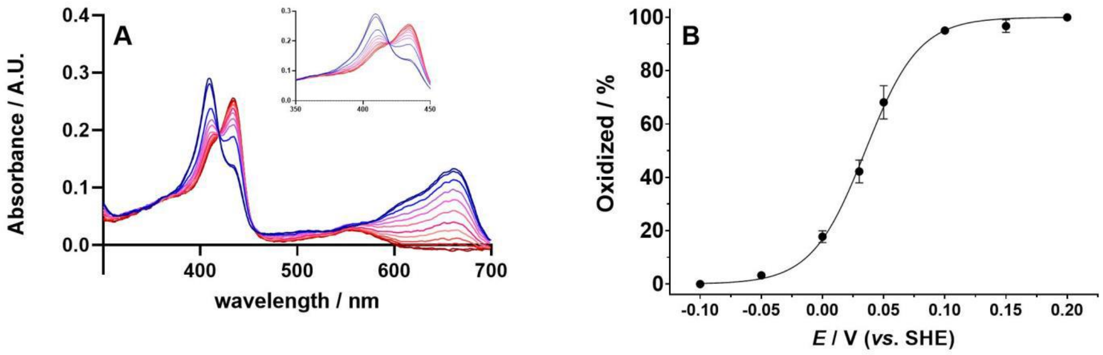

Figure S7. Redox potential measurement of FePPIX-Mb

Figure A shows two step spectroelectrochemistry of 3.5 μM FePPIX–Mb. The spectrum shows the change in absorbance from oxidized (dark blue line) to reduced (red line), reporting the absorbance recorded after potential steps (50 mV) every 120 seconds. The change in absorbance at 406 nm was followed and fitted to the Nernst equation to obtain *E*0' = +0.054 ± 0.004 V vs SHE, where *n* = 1.17 ± 0.16. (B) Percentage of oxidized species of FePPIX–Mb in respect to the change of potential following the absorbance at 406 nm.

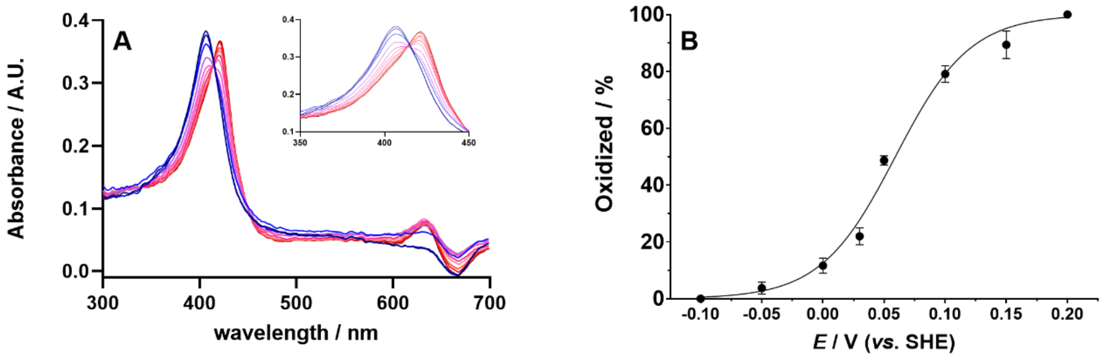

Figure S8. Redox potential measurement of FeCe6-Mb

Figure A shows two step spectroelectrochemistry of 3.5 μM FeCe6–Mb. The spectrum shows the change in absorbance from oxidized (dark blue line) to reduced (red line), reporting the absorbance recorded after potential steps (50 mV) every 120 seconds. A_{max} = 0.38 at 410 nm (λ_{max}) with an extinction coefficient (Ɛ) = 109,830 M^{-1} cm^{-1}. The change in absorbance at 410 nm was followed and fitted to the Nernst equation to obtain $E0'$ = +0.074 ± 0.003 V vs SHE, where n = 0.85 ± 0.16. (B) Percentage of oxidized species of FeCe6–Mb in respect to the change of potential following the absorbance at 410 nm.

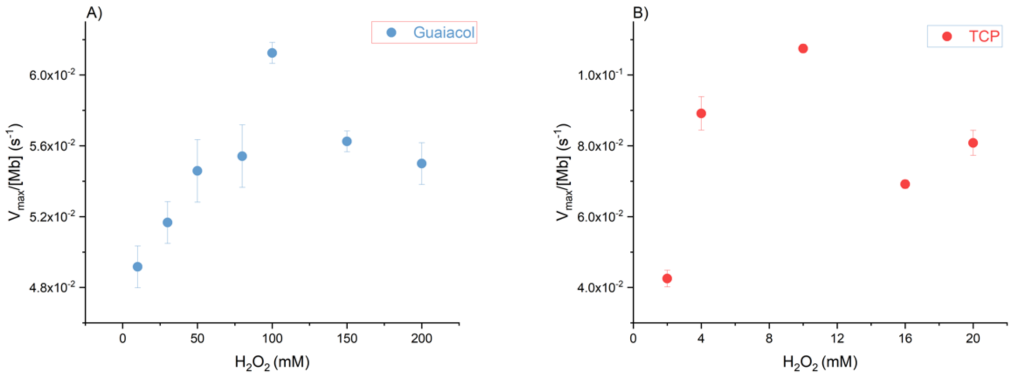

Figure S9. Peroxidase activity of FePPIX-Mb with varied concentration of H_2O_2

Figure A) shows the V_0 (initial rate) of FePPIX-Mb with varied [H_2O_2] when the [Mb] was 2 μM and [guaiacol] was 5 mM. B) shows the V_0 of FePPIX-Mb with varied [H_2O_2] when the [Mb] was 1 μM and [TCP] was 1 mM. All the reactions were carried out at 22 °C in PBS buffer (pH 7.4).

Table S4. Summary of screening Mb variants on guaiacol and TCP oxidation

Enzyme	Guaiacol Oxidation				TCP Oxidation			
	[Mb]	[Guaiacol]	[H_2O_2]	V_0/[Mb]	[Mb]	[TCP]	[H_2O_2]	V_0/[Mb]
FePPIX-Mb	2 µM	5 mM	100 mM	0.06 ± 0.0006	1 µM	1 mM	10 mM	0.057 ± 0.003
CuPPIX-Mb	2 µM	5 mM	100 mM	-[a]	1 µM	1 mM	10 mM	-[a]
CoPPIX-Mb	2 µM	5 mM	100 mM	-[a]	1 µM	1 mM	10 mM	-[a]
MnPPIX-Mb	2 µM	5 mM	100 mM	-[a]	1 µM	1 mM	10 mM	0.066 ± 0.002
NiPPIX-Mb	2 µM	5 mM	100 mM	-[a]	1 µM	1 mM	10 mM	-[a]
RuPPIX-Mb	2 µM	5 mM	100 mM	-[a]	1 µM	1 mM	10 mM	-[a]
FeCe6-Mb	2 µM	5 mM	100 mM	0.054 ± 0.0024	1 µM	1 mM	10 mM	0.092 ± 0.002
CuCe6-Mb	3 µM	10 mM	100 mM	0.002 ± 0.0004	1 µM	1 mM	10 mM	0.013 ± 0.001
CoCe6-Mb	2 µM	5 mM	100 mM	-[a]	1 µM	1 mM	10 mM	-[a]
MnCe6-Mb	1 µM	5 mM	100 mM	0.048 ± 0.0012	1 µM	1 mM	10 mM	0.081 ± 0.005
NiCe6-Mb	2 µM	5 mM	100 mM	-[a]	1 µM	1 mM	10 mM	-[a]
RuCe6-Mb	2 µM	5 mM	100 mM	-[a]	1 µM	1 mM	10 mM	-[a]

[a] No conversion.

Mean values and standard deviations of triplicates are shown.

Table S5 Reaction conditions of steady state kinetic measurement

	FePPIX-Mb (2 µM)	FeCe6-Mb (2 µM)	CuCe6-Mb (3 µM)	MnCe6-Mb (1 µM)	MnPPIX-Mb
Guaiacol (mM)	4 – 20	1 – 30	10 – 80	2 – 10	-[a]
H_2O_2 (mM)	100				
	FePPIX-Mb (0.5 µM)	FeCe6-Mb (1 µM)	CuCe6-Mb (1 µM)	MnCe6-Mb (1 µM)	MnPPIX-Mb (1 µM)
TCP (mM)	0.25 – 3	0.1 – 2.5	0.1 – 2	0.1 – 1.5	0.1 – 1.5
H_2O_2 (mM)	10				

[a] No reaction was detected

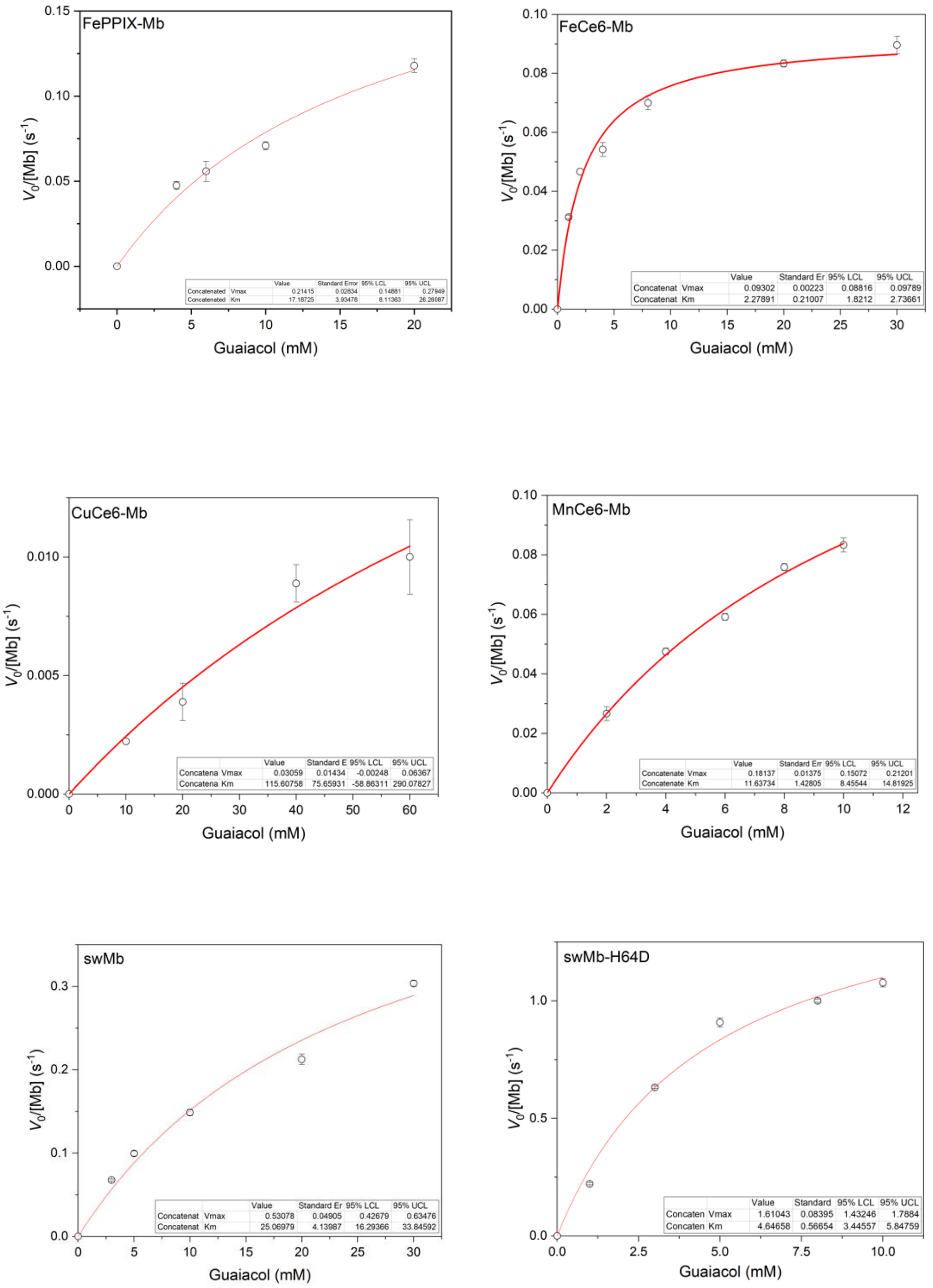
FePPIX-Mb
V0/[Mb] (s-1)
Guaiacol (mM)
Value Standard Error 95% LCL 95% UCL
Concatenated Vmax 0.21415 0.02834 0.14881 0.27949
Concatenated Km 17.18725 3.93478 8.11363 26.26087
FeCe6-Mb
V0/[Mb] (s-1)
Guaiacol (mM)
Value Standard Er 95% LCL 95% UCL
Concatenat Vmax 0.09302 0.00223 0.08816 0.09789
Concatenat Km 2.27891 0.21007 1.8212 2.73661
CuCe6-Mb
V0/[Mb] (s-1)
Guaiacol (mM)
Value Standard E 95% LCL 95% UCL
Concatena Vmax 0.03059 0.01434 -0.00248 0.06367
Concatena Km 115.60758 75.65931 -58.86311 290.07827
MnCe6-Mb
V0/[Mb] (s-1)
Guaiacol (mM)
Value Standard Err 95% LCL 95% UCL
Concatenate Vmax 0.18137 0.01375 0.15072 0.21201
Concatenate Km 11.63734 1.42805 8.45544 14.81925
swMb
V0/[Mb] (s-1)
Guaiacol (mM)
Value Standard Er 95% LCL 95% UCL
Concatenat Vmax 0.53078 0.04905 0.42679 0.63476
Concatenat Km 25.06979 4.13987 16.29366 33.84592
swMb-H64D
V0/[Mb] (s-1)
Guaiacol (mM)
Value Standard 95% LCL 95% UCL
Concaten Vmax 1.61043 0.08395 1.43246 1.7884
Concaten Km 4.64658 0.56654 3.44557 5.84759

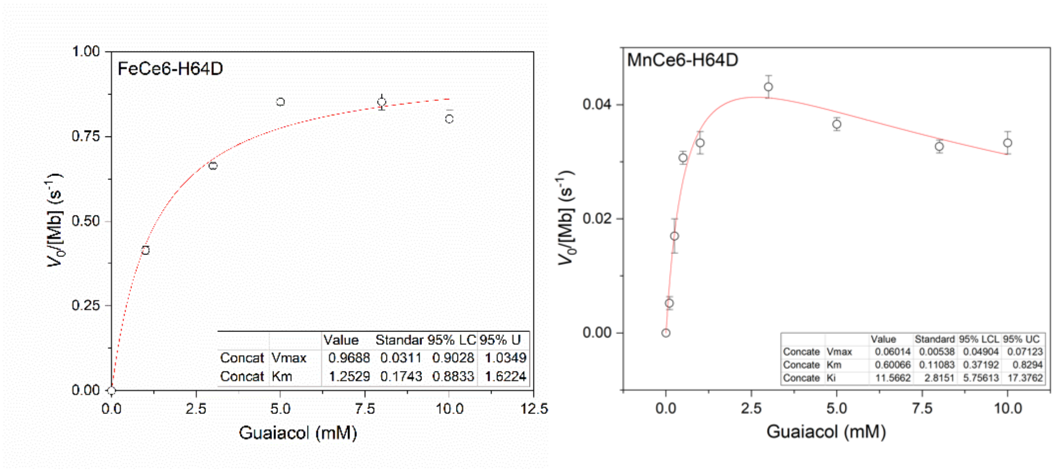

Figure S10. Plots of initial rates for Mb variants-catalysed guaiacol oxidation reactions

Figure shows the plots of initial rate (V_0/[Mb]) *versus* [guaiacol] of peroxidase reactions catalysed by FePPIX-Mb, FeCe6-Mb, CuCe6-Mb and MnCe6-Mb, separately. The simulated steady stare kinetics were obtained from the curve by applying the Michaelis-Menten equation. Guaiacol's oxidation product was measured based on its absorbance at 470 nm. The measurement was carried out at 22 °C in PBS buffer (pH 7.4). Means and standard deviations of triplicates are shown.

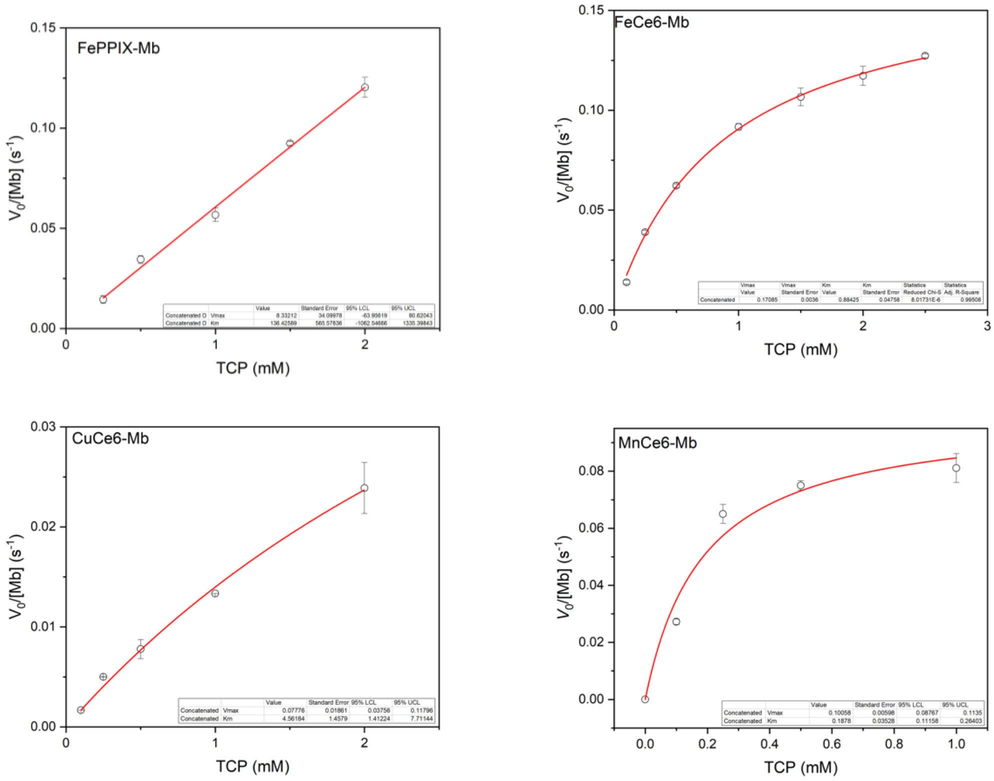

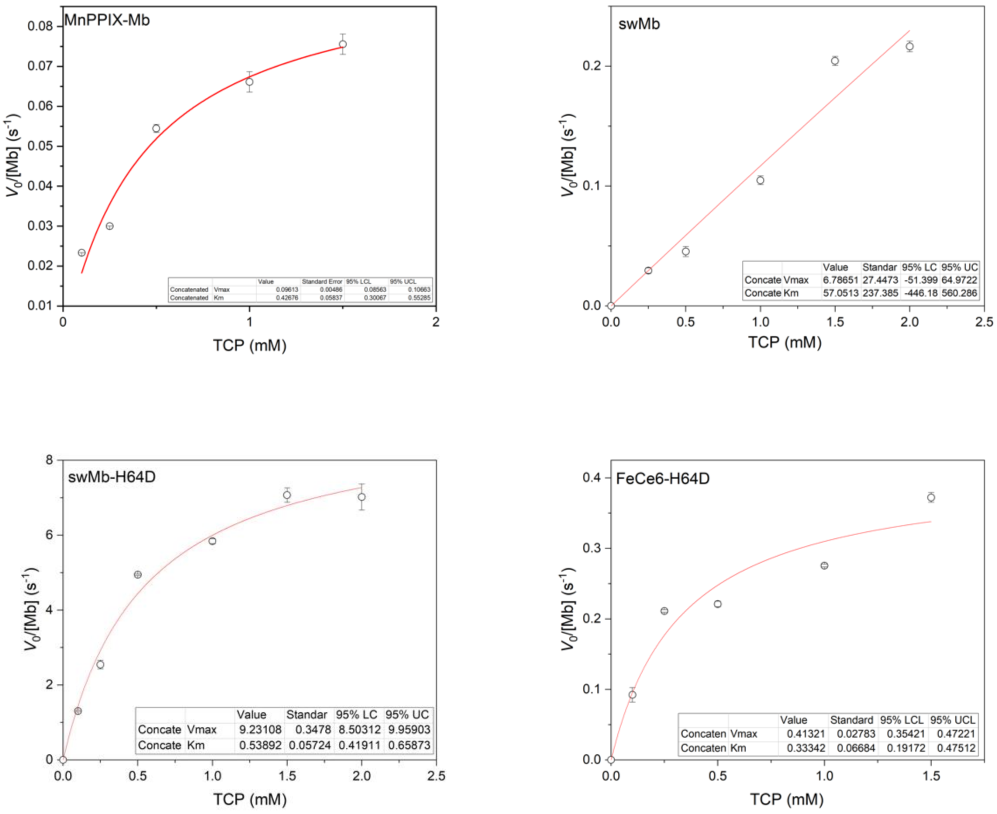

Figure S11. Plots of initial rates for Mb variants-catalysed TCP dehalogenation reactions

Figure shows the plots of initial rate (V_0/[Mb]) *versus* [TCP] of peroxidase reactions catalysed by FePPIX-Mb, FeCe6-Mb, CuCe6-Mb, MnCe6-Mb and MnPPIX-Mb, separately. The simulated steady stare kinetics were obtained from the curve by applying the Michaelis-Menten equation. TCP's oxidation product was measured based on its absorbance at 272 nm. The measurement was carried out at 22 °C in PBS buffer (pH 7.4). Mean values and standard deviations of triplicates are shown.

Table S6. Peroxidase activity of reconstituted wild-type Mb (FePPIX-Mb) and of artificial cofactor Mb variants: Kinetic parameters for H_2O_2-dependent oxidation of guaiacol and TCP catalysed by Mb and its variants.

Enzyme	Guaiacol			TCP		
	k_{cat} (s^{-1})	K_m (mM)	k_{cat}/K_m ($M^{-1}\bullet s^{-1}$)	k_{cat} (s^{-1})	K_m (mM)	k_{cat}/K_m ($M^{-1}\bullet s^{-1}$)
FePPIX-Mb	0.21 ± 0.028	17.19 ± 3.93	12.46 ± 3.29	8.33 ± 34.10	136.43 ± 565	61.07 ± 355.8
FeCe6-Mb	0.09 ± 0.002	2.28 ± 0.21	40.82 ± 3.89	0.17 ± 0.004	0.88 ± 0.048	193.21 ± 11.17
CuCe6-Mb	0.03 ± 0.014	115.60 ± 75.66	0.26 ± 0.21	0.08 ± 0.019	4.56 ± 1.46	17.05 ± 6.81
MnCe6-Mb	0.18 ± 0.014	11.64 ± 1.43	15.59 ± 2.25	0.10 ± 0.006	0.19 ± 0.035	535.57 ± 105.5
MnPPIX-Mb	-[a]	-[a]	-[a]	0.096 ± 0.005	0.43 ± 0.058	225.26 ± 32.85
swMb	0.53 ± 0.05	25.07 ± 4.14	21.18 ± 4.01	6.79 ± 27.45	57.05 ± 237	118.95 ± 690
swMb-H64D	1.61± 0.08	4.65 ± 0.56	346.58 ± 45.96	9.23 ± 0.35	0.54 ± 0.057	17100 ± 1930
FeCe6-swMb-H64D	0.97 ± 0.03	1.25 ± 0.17	773.32 ± 110.44	0.41± 0.027	0.33 ± 0.067	690.25 ± 262.0
MnCe6-swMb-H64D	0.06 ± 0.005	0.60 ± 0.11	100.12 ± 20.53	-[a]	-[a]	-[a]

[a] No conversion.

Reaction conditions: guaiacol oxidation, 100 mM H_2O_2 and variable [guaiacol]. TCP oxidation, 10 mM H_2O_2 and variable [TCP]. All reactions were carried out in PBS buffer (pH 7.4) at 22 °C. Mean values and standard deviations of triplicates are shown.

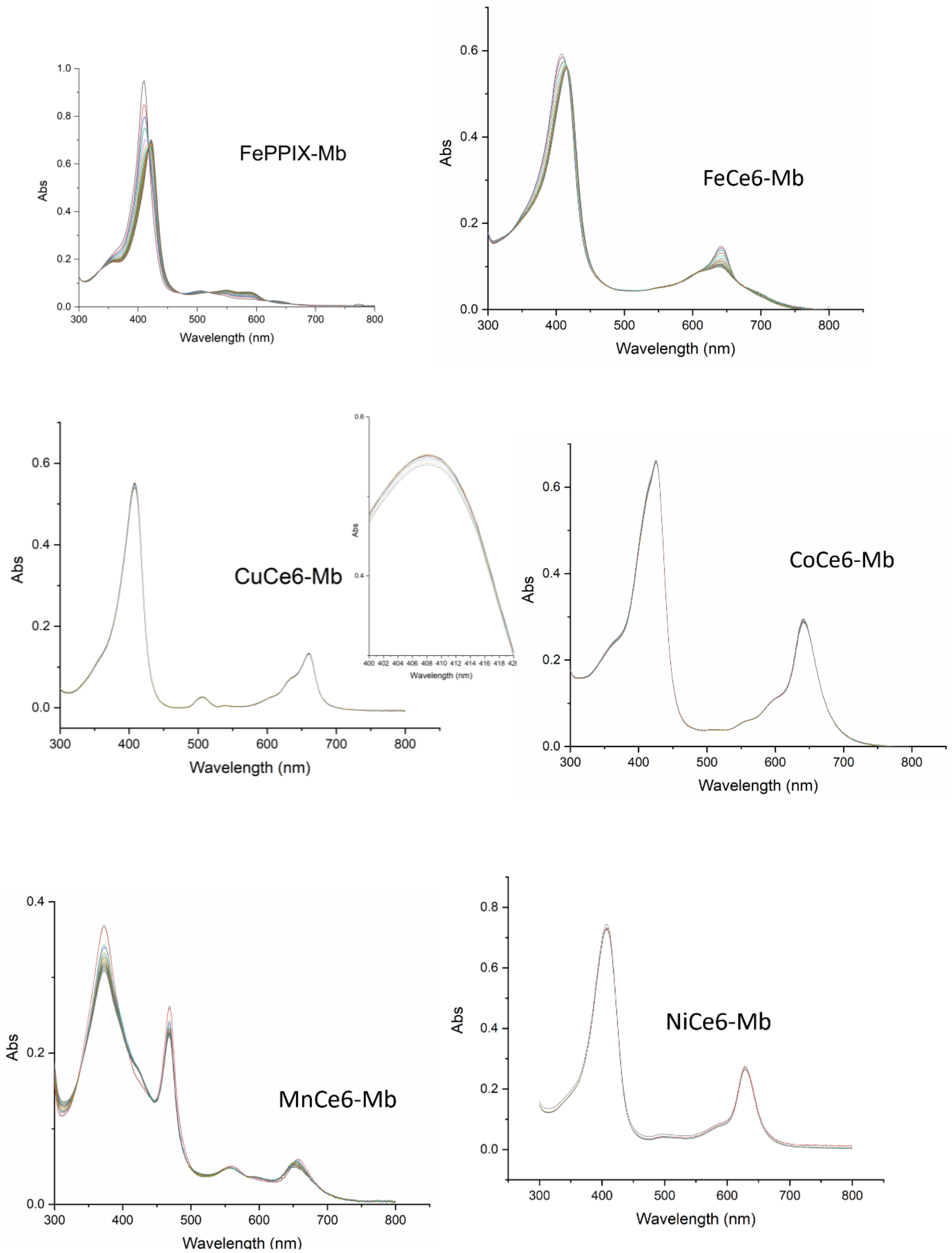
FePPIX-Mb
FeCe6-Mb
CuCe6-Mb
CoCe6-Mb
MnCe6-Mb
NiCe6-Mb
Abs
Wavelength (nm)

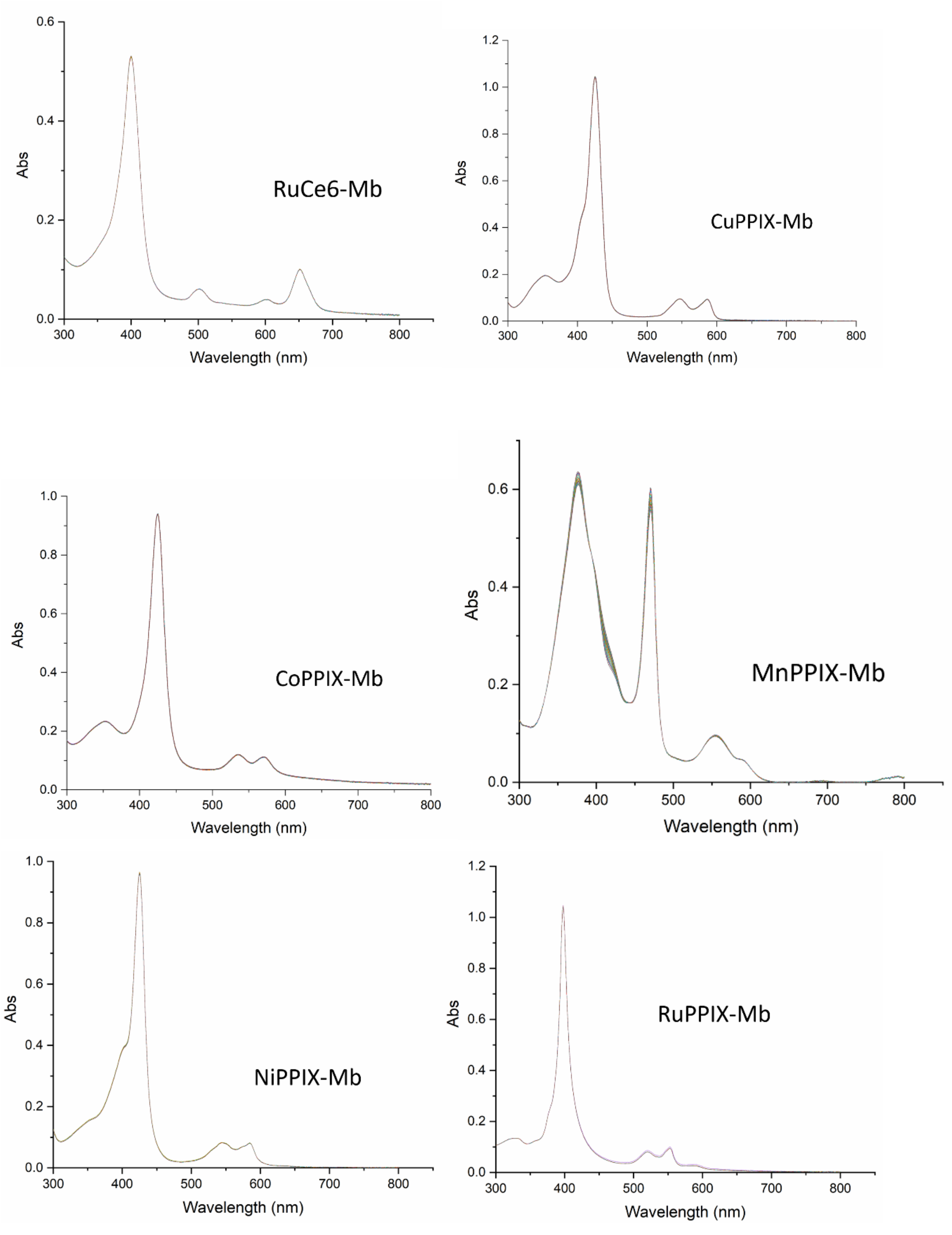

Figure S12. UV-Vis spectra of Mb variants reacted with H_2O_2

Figure shows overlayed UV-Vis spectra when 0.01 mM Mb and its variants were reacted with 40 equiv. H_2O_2 in PBS buffer (pH 7.4) at 22 °C. Spectra were recorded every 0.1 s.

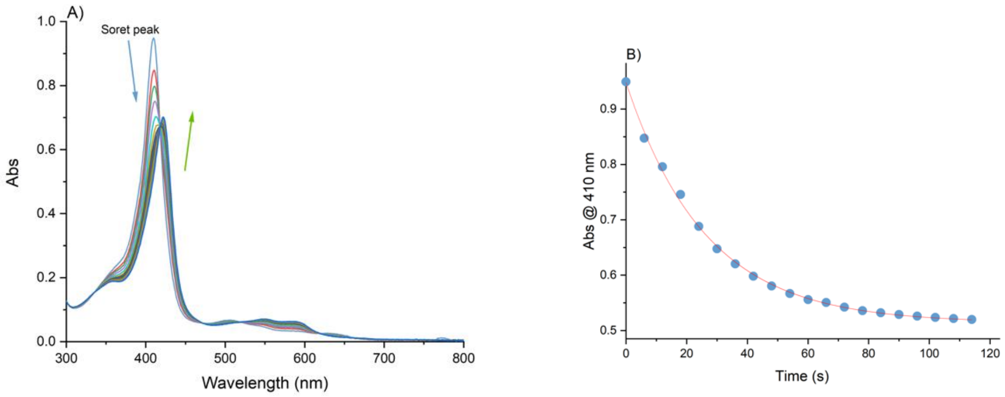

Figure S13. Measurement of compound II formation rate

Figure A) shows overlayed UV-Vis spectra when FePPIX-Mb react with 40 equiv. H_2O_2. B) shows the single exponential fit for the decay of Soret peak of FePPIX-Mb with 40 equiv. H_2O_2.

Table S7. Summary of $1/k_{obs}$ of Mb variants reacted with H_2O_2

Enzyme	$1/k_{obs}$							
[H_2O_2]	40x	SD	20x	SD	10x	SD	5x	SD
FePPIX-Mb	13.60	0.016	28.80	0.043	49.82	0.090	86.61	0.27
CuPPIX-Mb	-a	-a	-a	-a	-a	-a	-a	-a
CoPPIX-Mb	-a	-a	-a	-a	-a	-a	-a	-a
MnPPIX-Mb	138.03	4.97	100.1	6.11	178.34	29.56	243.5	105.4
NiPPIX-Mb	-a	-a	-a	-a	-a	-a	-a	-a
RuPPIX-Mb	-a	-a	-a	-a	-a	-a	-a	-a
FeCe6-Mb	20.01	0.15	29.24	0.21	38.32	0.25	53.62	0.30
CuCe6-Mb	36.73	4.18	61.61	11.27	94.80	31.62	117.81	77.01
CoCe6-Mb	-a	-a	-a	-a	-a	-a	-a	-a
MnCe6-Mb	33.85	0.26	43.66	0.39	74.90	0.81	137.33	7.58
NiCe6-Mb	-a	-a	-a	-a	-a	-a	-a	-a
RuCe6-Mb	-a	-a	-a	-a	-a	-a	-a	-a

[a] No conversion.

0.14 mM Mb and its variants reacted with 40, 20, 10, 5 equiv. H_2O_2 (5.6, 2.8, 1.4 and 0.7 mM) in PBS buffer (pH 7.4) at 22 °C.

$1/k_{obs}$ was directly obtained from the single exponential fit of the Soret decay.

Mean values and standard deviations of triplicates are shown.

3. References

[1] N. Bruns, K. Pustelny, L. M. Bergeron, T. A. Whitehead, D. S. Clark, *Angew. Chem., Int. Ed.* **2009**, *48*, 5666-5669.

[2] M. R. Green, J. Sambrook, *Cold Spring Harbor Protocols* **2018**, *2018*, pdb. prot101188.

[3] a) *Cold Spring Harbor Protocols* **2006**, *2006*, pdb.rec8303; b) *Cold Spring Harbor Protocols* **2018**, pdb.rec099085.

[4] G. Sreenilayam, E. J. Moore, V. Steck, R. Fasan, *ACS Catalysis* **2017**, *7*, 7629-7633.

[5] F. W. J. Teale, *Biochim. Biophys. Acta* **1959**, *35*, 543.

[6] a) A. J. Miles, S. G. Ramalli, B. A. Wallace, *Protein Sci.* **2021**, *31*, 37-46; b) L. Whitmore, B. A. Wallace, *Biopolymers: Original Research on Biomolecules* **2008**, *89*, 392-400.

[7] R. J. Simpson, *Cold Spring Harbor Protocols* **2007**, *2007*, pdb.prot4719.

[8] K. K. Khan, M. S. Mondal, L. Padhy, S. Mitra, *Europ. J. Biochem.* **1998**, *257*, 547-555.

[9] M. Pott, T. Hayashi, T. Mori, P. R. E. Mittl, A. P. Green, D. Hilvert, *J. Am. Chem. Soc.* **2018**, *140*, 1535-1543.

[10] C.-z. Li, K. Nishiyama, I. Taniguchi, *Electrochim. Acta* **2000**, *45*, 2883-2888.

[11] I. Taniguchi, C.-z. Li, M. Ishida, Q. Yao, *J. Electroanal. Chem.* **1999**, *460*, 245-250.

[12] T. S. Srivastava, *Biochim. Biophys. Acta, Protein Struct.* **1977**, *491*, 599-604.

[13] G. Sreenilayam, E. J. Moore, V. Steck, R. Fasan, *ACS Catal.* **2017**, *7*, 7629-7633.
